# β-Agonists Selectively Modulate Proinflammatory Gene Expression in Skeletal Muscle Cells via Non-Canonical Nuclear Crosstalk Mechanisms

**DOI:** 10.1371/journal.pone.0090649

**Published:** 2014-03-06

**Authors:** Krzysztof Kolmus, Marleen Van Troys, Karlien Van Wesemael, Christophe Ampe, Guy Haegeman, Jan Tavernier, Sarah Gerlo

**Affiliations:** 1 Department of Medical Protein Research, VIB, Gent, Belgium; 2 Department of Biochemistry, Faculty of Medicine and Health Sciences, Ghent University, Gent, Belgium; 3 Department of Physiology, Faculty of Sciences, Ghent University, Gent, Belgium; University of Texas Health Science Center at Houston, United States of America

## Abstract

The proinflammatory cytokine Tumour Necrosis Factor (TNF)-α is implicated in a variety of skeletal muscle pathologies. Here, we have investigated how *in vitro* cotreatment of skeletal muscle C2C12 cells with β-agonists modulates the TNF-α-induced inflammatory program. We observed that C2C12 myotubes express functional TNF receptor 1 (TNF-R1) and β2-adrenoreceptors (β2-ARs). TNF-α activated the canonical Nuclear Factor-κB (NF-κB) pathway and Mitogen-Activated Protein Kinases (MAPKs), culminating in potent induction of NF-κB-dependent proinflammatory genes. Cotreatment with the β-agonist isoproterenol potentiated the expression of inflammatory mediators, including Interleukin-6 (IL-6) and several chemokines. The enhanced production of chemotactic factors upon TNF-α/isoproterenol cotreatment was also suggested by the results from migrational analysis. Whereas we could not explain our observations by cytoplasmic crosstalk, we found that TNF-R1-and β2-AR-induced signalling cascades cooperate in the nucleus. Using the IL-6 promoter as a model, we demonstrated that TNF-α/isoproterenol cotreatment provoked phosphorylation of histone H3 at serine 10, concomitant with enhanced promoter accessibility and recruitment of the NF-κB p65 subunit, cAMP-response element-binding protein (CREB), CREB-binding protein (CBP) and RNA polymerase II. In summary, we show that β-agonists potentiate TNF-α action, via nuclear crosstalk, that promotes chromatin relaxation at selected gene promoters. Our data warrant further study into the mode of action of β-agonists and urge for caution in their use as therapeutic agents for muscular disorders.

## Introduction

Skeletal muscle atrophy is a devastating consequence of a large number of diseases, including cancer and myopathies, but is also apparent in physiological processes, such as aging or disuse. Several lines of evidence indicate that inflammatory factors contribute to the loss of skeletal muscle mass and function [1]. One of the cytokines that has been especially associated with the development of skeletal muscle abnormalities is Tumour Necrosis Factor (TNF)-α and elevated levels of TNF-α are apparent in skeletal muscle wasting disorders [2]. TNF-α transduces its activity via two different types of membrane-bound receptors, namely TNF-receptor 1 (TNF-R1) and TNF-receptor 2 (TNF-R2), which stimulate different cellular processes. TNF-R ligation leads to the recruitment of receptor-specific adaptor proteins, which in turn activate a cascade of protein kinases and several downstream transcription factors, including the Nuclear Factor (NF)- κB [3].

NF-κB is the generic term for members of a family of ubiquitously expressed transcription factors, that act as homo- or heterodimers to regulate genes involved in immunity and inflammation [4]. In the context of inflammatory gene expression, the p65-p50 NF-κB heterodimer has been most intensively studied. TNF-α induces the canonical NF-κB signalling pathway, marked by activation of the IκB kinase β (IKKβ) complex, which phosphorylates the IκBα inhibitor proteins that, in resting cells, sequester NF-κB in the cytoplasm. Phosphorylated IκBα is ubiquitinylated and targeted for proteasomal degradation, allowing NF-κB to migrate from the cytoplasm to the nucleus, where it drives transcription of genes containing NF-κB-responsive elements [4]. Whereas NF-κB function has been mainly studied in immune cells, recent reports have demonstrated a role for NF-κB in a variety of other cell types, including skeletal muscle. For instance, it was shown that interference with NF-κB activity, via overexpression of IκB supersuppressor or p65 knock-out, reduces inflammation and improves the regeneration process in different skeletal muscle disease models [1], [5].

The adrenergic receptors belong to the family of G-protein coupled receptors (GPCRs) and skeletal muscle cells express mainly the β_2_-adrenoreceptor (β_2_-AR) subtype [6]. β_2_-AR agonists (β-agonists) are well known for their anabolic properties and several *in vivo* studies support the therapeutic potential of β-agonists in skeletal muscle wasting disorders [7]-[9]. Interestingly, the existence of extensive crosstalk between β_2_-AR and TNF-R-mediated signalling cascades was documented in different cell types and it was postulated that β-agonists have anti-inflammatory effects, that can be, at least in part, explained by inhibition of NF-κB activity [10]–[12]. Arguing against the anti-inflammatory effects of β_2_-AR stimulation is the repeated observation, in different model systems, that β-agonists potentiate the expression of the prototypical inflammatory cytokine IL-6, a phenomenon that was also reported in skeletal muscle cells *in vivo* and *in vitro* [13], [14].

Recent genome-wide expression studies have yielded detailed information on the individual effects of TNF-α or β-agonists on the skeletal muscle transcriptome [15], [16]. However, the effect of controlled cotreatment of skeletal muscle cells with TNF-α and β-agonists has, to our knowledge, never been investigated. In addition, the molecular basis for the reported selective effect of β-agonists on NF-κB dependent gene expression remains to be elucidated. Therefore, we have investigated how β-agonists modulate TNF-α-induced signalling cascades, focusing on the NF-κB pathway and its target genes in C2C12 myotubes. As opposed to previous reports in other cell types, we found no direct inhibitory effects of β-agonists on the NF-κB cascade in C2C12 skeletal muscle cells. Instead, we found that the β-agonist isoproterenol potently enhanced TNF-α-induced expression of selected NF-κB target genes, such as interleukin-6 (IL-6) and chemokine (C-X-C motif) ligand 5 (CXCL5). In addition, we report that this selective potentiation is not mediated by cytoplasmic modulation of NF-κB function, but instead relies on atypical epigenetic events. Finally, in support of the physiological relevance of our findings, the co-activation of β_2_-AR/TNF-Rs in myotubes promoted the migration of undifferentiated myoblasts.

## Materials and Methods

### Reagents and Antibodies

Isoproterenol and Trichostatin A (TSA) were purchased from Sigma Aldrich (St. Louis, MO, USA) and used at 10 µM and 100 nM, respectively. Murine TNF-α was a gift from the VIB Department for Molecular Biomedical Research of Ghent University (VIB-UGent, Gent, Belgium) and was used at 2000 IU/ml. Insulin-like growth factor-1 (IGF-1) was from ImmunoTools (Friesoythe, Germany) and was used at 10 ng/ml. Anti-β_2_-AR, anti-TNF-R1, anti-myogenin, anti-PARP, anti-P-H3-Ser10, anti-CBP, anti-RNA polymerase II, anti-p65, anti-IκBα, anti-P-CREB-Ser133 and anti-PKAc were from Santa Cruz Biotechnology (Santa Cruz, CA, USA). Anti-P-p65-Ser536, anti-P-ERK-Thr202/Tyr204, anti-P-JNK-Thr183/Tyr185, anti-P-p38-Thr180/Tyr182, anti-P-MSK-1-Thr581, anti-Lamin A/C and anti-CREB were from Cell Signaling Technology (Danvers, MA, USA). Anti-Ac-H3-Lys27 and GAPDH were from AbCam (Cambridge, UK). Anti-α-tubulin and anti-α-actin were from Sigma-Aldrich. In figures, the expression “antibody anti-” was substituted by the Greek letter “α-”. AatII and HincII restriction enzymes were obtained from New England BioLabs (Ipswich, MA, USA).

### Cell culture

Murine C2C12 cells (European Collection of Cell Cultures, Salisbury, UK) were maintained in Dulbecco's Modified Eagle's Medium (DMEM, Gibco by Life Technologies) supplemented with 10% foetal bovine serum (Gibco by Invitrogen, Paisley, UK), 100 IU/ml penicillin and streptomycin (Gibco by Life Technologies, Grand Island, NY, USA) (referred to herein after as growth medium or GM). Cells were cultured in an incubator at 37°C in a humidified atmosphere containing 5% CO_2_. Cells were passaged using 0.05% Trypsin+EDTA (Gibco by Life Technologies). In experiments using myoblasts, cells were seeded at 200000 cells/well in a 6-well format and GM was substituted by starvation medium (DMEM containing 1% foetal bovine serum, 100 IU/mL penicillin and streptomycin, referred as SM) for 24 hours. In experiments using myotubes, cells were seeded at 300000 cells/well in a 6-well format and GM was substituted by differentiation medium to induce differentiation (DMEM containing 2% horse serum, 100 U/ml penicillin and streptomycin, referred as differentiation medium or DM). To obtain myotubes, cells were cultured for 5 days in DM.

### RNA isolation, cDNA synthesis and Real-Time Quantitative PCR (RT-qPCR)

Total RNA was extracted with the RNeasy Mini Kit (Qiagen, Venlo, Netherlands). Reverse transcription was performed on 0.5 µg of total mRNA using the PrimeScript RT reagent kit from Takara Bio Inc. (Shiga, Japan). For real time cDNA amplification we used the Roche SYBR Green Mastermix (Roche Applied Science, Penzberg, Germany) and primers listed in Table S1. Fluorescence was monitored using the Light Cycler 480II (Roche). A serial dilution of a representative cDNA sample was used to generate a standard curve and determine the efficiency of the PCR reaction for all primer sets which was used in the subsequent calculation of relative mRNA inputs. Expression of each gene was normalized to expression of the HPRT house-keeping gene and results are presented as fold induction compared to untreated cells. For clarity the Y-axis is interrupted in some cases.

### Luciferase reporter assay

The NF-κB-luciferase reporter plasmid and the β-galactosidase control constructs have been described elsewhere [17]. The CRE-luciferase reporter plasmid was from Stratagene (LaJolla, CA). Transient transfection was performed using the Neon Transfection system (Invitrogen), according to the manufacturer's instructions for the murine C2C12 cell line. Briefly, cells were transfected with 1 µg of luciferase reporter constructs harbouring a consensus NF-κB site or CREB binding site and seeded to semi-confluence in 24-well plates. Cells were cotransfected with a β-galactosidase reporter construct (200 ng) containing the constitutively active pPGK promoter to correct for transfection efficiency. Following transfection, cells were differentiated for 5 days. Subsequently, cells were induced with isoproterenol and/or TNF-α for 6 hours. Total lysate was then incubated with luciferase substrate and luminescence was measured on a TopCount luminometer (PerkinElmer Life Sciences, Canberra-Packard, Waverley, UK). Luciferase activity was expressed as fold induction (treated/untreated) upon normalisation for transfection efficiency.

### Isolation of cellular proteins

For preparation of total protein lysates, cells were washed with ice-cold Phosphate Buffered Saline (PBS) and subsequently lysed in RIPA lysis buffer (200 mM NaCl, 50 mM Tris HCl pH 8.0, 0.05% SDS, 2 mM EDTA, 1% NP40, 0.5% Na-Deoxycholate, 2 mM Na_2_MoO_4_ and 10 mM NaF, Complete Protease Inhibitor Cocktail (Roche)) or in SDS sample buffer (10% glycerol, 62.5 mM Tris-HCl pH 6.8, 2% SDS, 0.5% β-mercaptoethanol and Bromophenol Blue).

For isolation of nuclear proteins, cells were washed with PBS, PBS with 2 mM Na_2_MoO_4_ and 10 mM NaF and buffer HB (20 mM Hepes pH 7.5, 10 mM NaF, 2 mM Na_2_MoO_4_, 0.2 mM EDTA pH 7.5). After aspiration of the washing buffer, lysis buffer (20 mM Hepes pH 7.5, 10 mM NaF, 2 mM Na_2_MoO_4_, 0.2 mM EDTA pH 7.5, 0.05% NP40, Complete Protease Inhibitor Cocktail (Roche)) was added. Subsequently, cells were scraped and collected. Samples were centrifuged at 17900 g for 1 minute in 4°C. The pellet was recovered and resuspended in Resuspension Buffer (20 mM Hepes pH 7.5, 0.1 mM EDTA pH 7.5, 5 mM NaF, 1 mM Na_2_MoO_4_, 20% glycerol) supplemented with the Complete Protease Inhibitor Cocktail (Roche). Next, equal volume of salt buffer (20 mM Hepes pH 7.5, 1.6 M NaCl, 0.1 mM EDTA pH 7.5, 5 mM NaF, 1 mM Na_2_MoO_4_, 20% glycerol, Complete Protease Inhibitor Cocktail (Roche)) was added. Samples were incubated for 30 minutes in the shaker at 4°C and subsequently centrifuged at 17900 g for 10 minutes at 4°C. Samples were next diluted to equal protein concentration (determined via BioRad protein assay) and 5x concentrated SDS sample buffer was added. The extracts were used subsequently for Western blotting analysis.

Protein contents of RIPA lysates and nuclear extracts were determined using the Bio-Rad protein assay according to manufacturer's instructions. Samples were next diluted to equal protein concentration and 5x concentrated SDS sample buffer was added. The extracts were used subsequently for Western blotting analysis.

### Western blotting

10 µg of nuclear proteins, 25 or 50 µg RIPA protein lysates or 25 µl of SDS sample buffer protein lysates were resolved using SDS-PAGE on 10% or 12% polyacrylamide gels, transferred to nitrocellulose membranes (Amersham, Dubendorf, Switzerland) and analysed by Western blotting. Briefly, membranes were incubated with Blocking Buffer (LICOR Biosciences, Lincoln, NE, USA) diluted with PBS in a 1∶1 ratio. Subsequently, membranes were probed with primary antibody diluted 1∶1000 in Blocking Buffer diluted 1∶1 with PBS containing 0.1% Tween (PBS-T). After washing in PBS-T, DyLight secondary antibody (Pierce, Rockford, IL, USA) was applied diluted 1∶10000 in Blocking Buffer/PBS-T (1∶1). The membranes were then washed in PBS-T and detection was performed using the Odyssey Imaging System (Licor). Subsequently, the membranes were reprobed with Actin, Tubulin, GAPDH, Lamin A/C or PARP antibodies to verify equal loading. Densitometric analysis averaging the results from independent Western blotting experiments was performed using ImageJ.

### Chromatin accessibility assay via Real-Time PCR (CHART-PCR)

The nuclease accessibility technique was performed as follows: cells were washed in PBS, scraped and collected by centrifugation at 453 g for 5 minutes at 4°C. Next, cells were resuspended in buffer A (10 mM Tris pH 7.5, 10 mM NaCl, 3 mM MgCl_2_, 0.3 M sucrose) and incubated for 10 minutes at 4°C. Next, equal volume of lysis buffer was added (10 mM Tris pH 7.5, 10 mM NaCl, 3 mM MgCl_2_, 0.3 M sucrose, 0.4% NP40 and 2 mM Na-butyrate) and cells were incubated for 10 minutes at 4°C. After centrifugation at 240 g for 5 minutes at 4°C, chromatin was resuspended in buffer R (10 mM Tris pH 7.5, 10 mM NaCl, 3 mM MgCl_2_, 1 mM Na-butyrate) digested for 30 min at 37°C with restriction enzymes in the buffer recommended by the manufacturer (New England BioLabs). Reactions were terminated by the addition of 2× Proteinase K buffer (100 mM Tris, pH 7.5, 200 mM NaCl, 2 mM EDTA, 1% SDS). Following proteinase K (Qiagen) and RNase A (Qiagen) treatment, genomic DNA (gDNA) was extracted using the phenol/chloroform/isoamyl alcohol (Sigma-Aldrich) method and subsequently resuspended in sterile water after ethanol precipitation. Purified gDNA (10 ng) was used for RT-PCR. Primers were designed to amplify sequences within the murine IL-6 promoter (Table S1). A serial dilution of a representative gDNA sample was used to generate a standard curve and determine the efficiency of the PCR reaction for all primer sets and to calculate the relative gDNA concentration ([gDNA]) of the samples. Data are presented as chromatin opening which was defined as the ratio of [gDNA] of samples digested with restriction enzymes over [gDNA] of undigested samples: Chromatin opening  =  [gDNA]digested/[gDNA]undigested.

### Chromatin immunoprecipitation (ChIP)

For ChIP experiments, protein/DNA complexes were crosslinked *in cellulo* by adding formaldehyde directly to the culture medium to a final concentration of 1%. After 10 minutes, glycine was added to a final concentration of 125 mM for another 10 minutes. Cells were washed, scraped and collected. Pellets were lysed in FA lysis buffer (0.1% SDS, 1% Triton X-100, 150 mM NaCl, 1 mM EDTA, 20 mM Tris pH 8.0) supplemented with Complete Protease Inhibitor Cocktail (Roche). Cells were sonicated using the Diagenode Bioruptor (Liège, Belgium) at high settings (8 min, 30 s on/30 s off, samples cooled on ice and repeat of the 8 minute cycle). Sonicated lysates were mixed in 1∶5 ratio with incubation buffer (0.15% SDS, 1% Triton X-100, 150 mM, 1 mM EDTA, 20 mM HEPES-KOH pH 7.5) followed by immunoprecipitation with 5 µg of anti-p65, anti-polymerase RNA II, anti-P-H3 Serine 10, anti-Ac-H3 Lys27, anti-CBP or anti-CREB using Protein A Sepharose 4 Fast Flow beads (Amersham). Samples were decrosslinked overnight at 65°C. All samples were treated with 50 µg/ml of RNase A and 100 µg/ml of Proteinase K. Immunoprecipitated genomic DNA (gDNA) was purified with the QiaQuick PCR purification kit (Qiagen) and subsequently quantified by Real Time PCR using the Roche SYBR Green Mastermix (Roche). Primers used for amplification of the IL-6 promoter and GAPDH control region are listed in Table S1. Quantitative PCR was performed on the Light Cycler 480II (Roche). Determination of [gDNA] in the input and immunoprecipitated samples (IPs) was performed as for CHART-PCR. Data are presented as the percentage (%) of [gDNA] in the IPs as compared to the [gDNA] in the corresponding input sample: %IP  =  ([gDNA]IP/[gDNA]input) ×100.

### Medium swap and migration assay

To investigate the influence of myotube-secreted factors on myoblast migration, a medium swap, followed by a migration assay, was performed. Conditioned media were prepared from untreated myotubes or myotubes treated with isoproterenol and/or TNF-α for 60 minutes. Subsequently, cells were washed twice with DMEM to remove the remaining inducers and incubated with serum-free DMEM for 24 hours to collect conditioned media with secreted factors. Conditioned media were filtered on 0.22 µm pore membranes (MILLEX GS, MF-Millipore MCE Membrane) and applied to myoblasts. As a positive control, IGF-1, dissolved in serum free DMEM, was used. Myoblasts were seeded in GM (20000 cells/well) on a collagen coating (25 µg/mL rat tail monomeric collagen type I; BD Biosciences, Two Oak Park, Bedford, MA, USA) in wells of a 96-well plate. A cell-free area was generated in the middle of the well using stoppers, according to the ORIS cell migration protocol (ORIS; Platypus Technologies). After 5 hours of cell adhesion, stoppers were removed, GM was aspirated, 100 µl of conditioned media was applied and cells were allowed to migrate for 24 hours into the cell-free central zone. Phase-contrast time-lapse movies were recorded for 24 hours with an interval of 10 minutes using a 10x UPlanFL objective (N.A. 0.30) on a CellM system with a IX81 microscope (Olympus). Four to eleven replicates were tested per condition and three independent experiments were performed. The area covered by the cell layer (that increases due to migration towards the central zone) was determined in time using customized software (CELLMIA, unpublished data). Cell migration velocity (mean of n replicates as indicated) is determined based on the slope of linear fit on the area over time data. The relative migration efficiency is the normalized cell velocity over different independent experiments.

### Statistical analysis

Statistical analysis was performed using Student's t-test or one-way ANOVA followed by Bonferroni's multiple comparison test with Graphpad Prism 4 software (Graphpad Software Inc.). Migration data are presented as mean ± standard error and statistical analysis was performed using Wilcoxon pairwise comparison with Bonferroni correction for multiple testing in R version 2.15.2 (The R Foundation for Statistical Computing). Results were considered significant when p-value <0.05.

## Results

### C2C12 myotubes express TNF-R1 and β2-ARs

**Figure 1:**
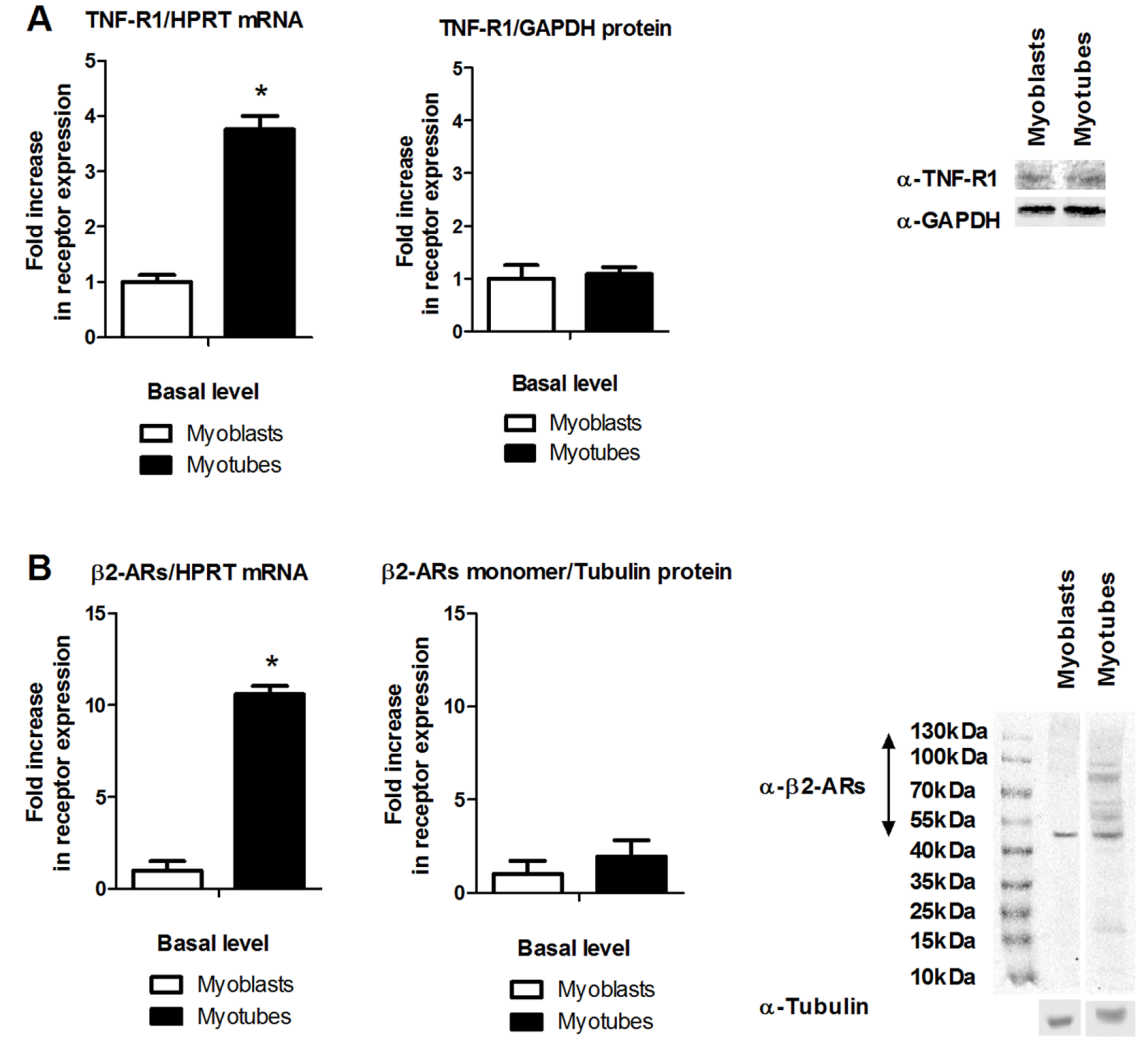
Expression of TNF-R1 and β_2_-AR in C2C12 myoblasts and myotubes. The basal expression of TNF-R1 (A) and β_2_-AR (B) mRNA and protein was compared in C2C12 myoblasts versus myotubes using RT-qPCR and Western blotting. RIPA lysates were prepared and equal amounts of protein were loaded on gel. Bar charts represent average ± SD of densitometric analysis of three independent experiments. (For the β_2_-AR Western blot only the monomeric form of the receptor was densitometrically quantified.) A representative blot is shown. (*) significantly different from myoblasts using student's t-test.

The murine C2C12 cell line is a well-established in vitro model for the study of molecular interactions occurring in skeletal muscle cells at different steps of myogenesis [18], [19]. We first investigated the expression levels of β2-ARs and TNF-R1/2 in undifferentiated myoblasts versus differentiated myotubes. Progression in myogenesis was confirmed by measuring myogenin expression (Figure S1A). We observed that myogenic differentiation was associated with augmented mRNA levels of TNF-R1 as well as β2-AR, as shown in Figure 1A and B, respectively. At the protein level, we did, however, not detect more TNF-R1 in myotubes as compared to myoblasts. Also, we did not detect an increase in the immunoreactive signal representing the monomeric β2-AR, which has a molecular weight of approximately 50 kDa. We did however observe that multiple high-molecular weight immunoreactive bands appear upon myogenic differentiation of C2C12 cells (Figure 1B). To corroborate that the high-molecular weight immunoreactivities indeed represent β2-ARs, we overexpressed a hemagglutinin (HA)-tagged β2-AR (β2-AR-HA) in HEK-293T cells, which do not express endogenous β2-AR. Like in C2C12 cells, we found higher molecular weight bands that react with the anti-β2-AR antibody. In addition, we found that these bands overlap with immunoreactivities detected with anti-HA, indicating they indeed derive from β2-AR species, and probably represent post-translationally modified or oligomeric forms of the β2-AR (Figure S1B) [20], [21]. In conclusion, these data indicate C2C12 myotubes express both TNF-R1 and β2-AR protein and indicate the expression of variant β2-AR species is upregulated in the course of myogenesis. Given that myotubes have been shown to secrete a wide variety of mediators when subjected to pro-inflammatory stimuli [22], and as C2C12 myotubes express both TNF-R1 and β2-AR2, we have used C2C12 myotubes for further experiments into β2-AR2/TNF-R1 crosstalk.

### Effect of β-agonist cotreatment on TNF-α-induced, NF-κB-dependent, inflammatory gene expression in skeletal muscle cells

In physiological and pathological circumstances, such as during an acute boost of exercise or in chronic inflammatory disease, skeletal muscle cells express a number of NF-κB-dependent cytokines and other inflammatory markers [1], [23]. Here, we performed a RT-qPCR analysis to assess how β-agonist cotreatment modulates the expression of well-known NF-κB target genes with a previously demonstrated role in skeletal muscle homeostasis [15], [23], [24]. The presence of NF-κB binding sites in the promoters of the selected genes was confirmed by a bioinformatics analysis using P-Scan (Table S2). For each gene, the promoter region of 500 bp upstream to the Transcription Starting Site was investigated with the Transcription Factor Binding Sites matrices available in the TRANSFAC databases.

**Figure 2:**
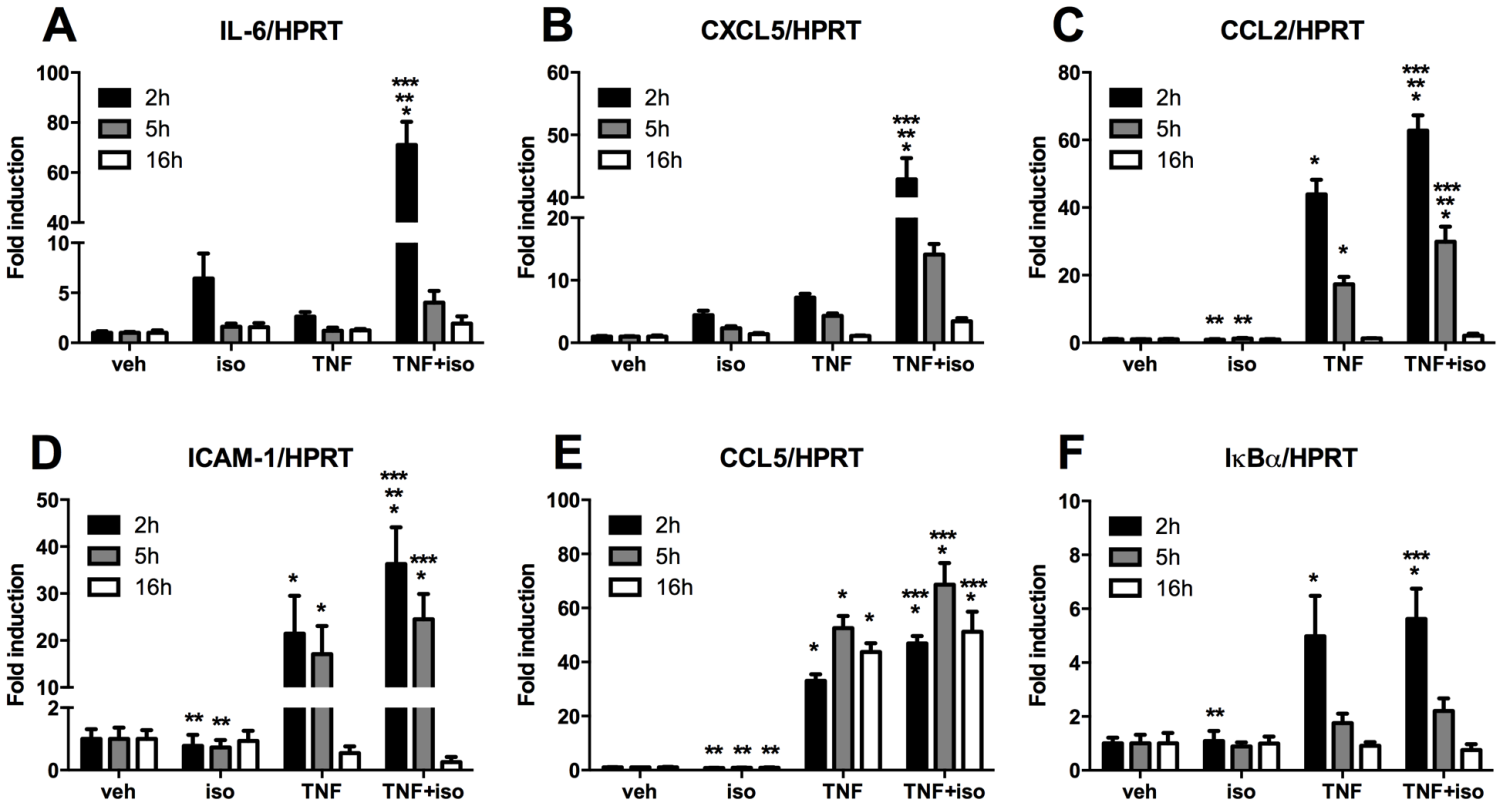
Effect of isoproterenol/TNF-α cotreatment on NF-κB-dependent gene expression in C2C12 myotubes. Expression of inflammatory markers was measured by RT-qPCR after 2, 5 and 16-hours induction with vehicle (veh), isoproterenol (iso) and/or TNF-α (TNF) in C2C12 myotubes. Fold induction for each gene was calculated versus veh control at the corresponding time point. Results represent average ± SD of three independent experiments. Statistical significance was determined via ANOVA followed by Bonferroni's multiple comparison test. (*) Significantly different from veh. (**) Significantly different from TNF. (***) Significantly different from iso.

We found that in C2C12 myotubes, TNF-α significantly induced the expression of intercellular adhesion molecule 1 (ICAM-1), NF-κB inhibitor alpha (IκBα), chemokine (C-C motif) ligand 2 (CCL2) and chemokine (C-C motif) ligand 5 (CCL5), whereas it had modest yet insignificant effects on the expression of interleukin-6 (IL-6) and chemokine (C-X-C motif) ligand 5 (CXCL5) (Figure 2). We found no clear effects of TNF-α on the expression of interleukin-7 (IL-7), interleukin-15 (IL-15) and Brain derived neurotrophic factor (BDNF) (Figure S2). Isoproterenol by itself did not significantly affect the expression of any of the investigated genes, but had a mild positive effect on the expression of CXCL5 and IL-6. Interestingly, we found that isoproterenol cotreatment did not significantly modulate TNF-α-induced CCL5 and IκBα transcription (Figure 2D, F), but enhanced the effect of TNF-α on the expression of CCL2 and ICAM-1 (Figure 2C, E) and induced a pronounced synergy for IL-6 and CXCL5 (Figure 2A, B, Table 1). Overall, the effect of TNF-α on the expression of the selected genes was rapid and transient, peaking after 2 hours of treatment and then rapidly declining, and these kinetics were not modulated by isoproterenol cotreatment. Only CCL5 mRNA levels peaked at 5 hours, both upon TNF-α and TNF-α and isoproterenol cotreatment, and were maintained even after 16 hours induction (Figure 2 D). We concluded that in C2C12 myotubes a subset of NF-κB-dependent genes exists with different responses to TNF-α or costimulation with isoproterenol.

**Table 1:** Synergy factors upon combined isoproterenol/TNF-α treatment in C2C12 skeletal muscle.

	Synergy Factor					
	2 h		5 h		16 h	
	Mean	St Dev	Mean	St Dev	Mean	St Dev
**IL-6**	8.80 (*)	3.13	1.48	0.74	0.68	0.31
**CXCL5**	3.98 (*)	1.20	2.07	0.51	1.38	0.37
**CCL2**	1.48	0.38	1.59	0.39	0.92	0.21
**CCL5**	1.52	0.44	1.22	0.28	1.14	0.30
**ICAM-1**	1.66	1.06	1.35	0.80	0.18	0.10
**IκBα**	0.93	0.46	0.77	0.29	0.40	0.16

The strength of the synergies was calculated by dividing the relative mRNA levels upon TNF-α/isoproterenol induction (Z) by the sum of the relative mRNA levels after individual TNF-α (X) and isoproterenol (Y) induction (i.e. Z/(X+Y)). (*) Significant synergy meaning Z/(X+Y) significantly larger than X+Y.

### Cytoplasmic signalling crosstalk upon β_2_-AR/TNF-R co-activation in C2C12 myotubes

**Figure 3:**
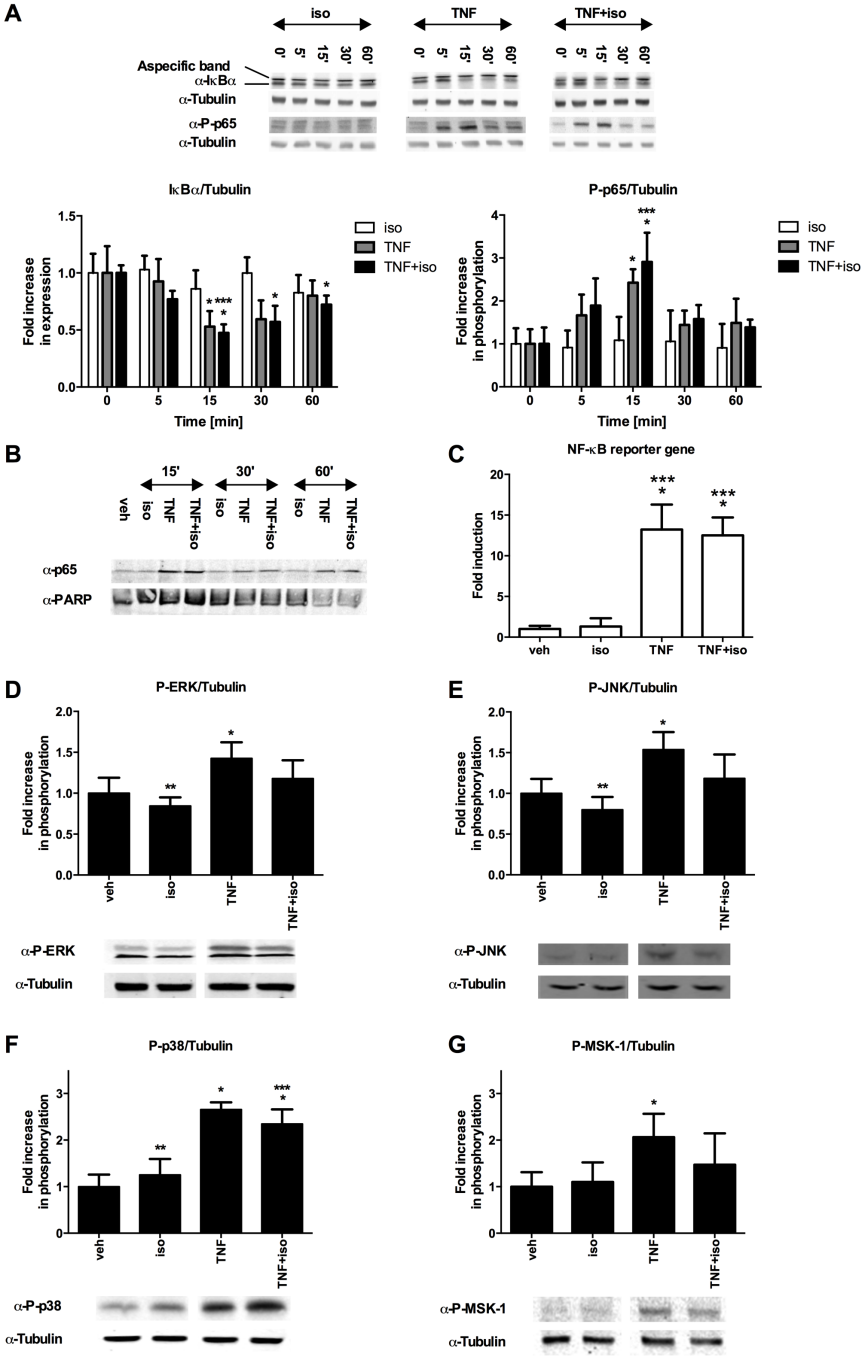
Effect of isoproterenol on TNF-α-induced NF-κB and MAPKs activation in C2C12 myotubes. (A) Kinetics of IκBα degradation and increase in phosphorylation of p65 at serine 536. After veh, iso and/or TNF treatment cells were lysed in SDS sample buffer and analysed via Western blotting. Bar charts represent average ± SD of densitometric analysis of three independent experiments. A representative blot is shown. (B) Kinetics of p65 nuclear translocation. After veh, iso and/or TNF treatment, nuclear extracts were prepared and analysed via Western blotting. A representative blot from two independent experiments is shown. (C) Induction of NF-κB reporter gene activity. C2C12 myotubes, transfected with the κB-luciferase reporter plasmid, were treated with veh, iso and/or TNF for 6 hours, before analysis of luciferase production. Fold induction was calculated versus veh control. Results represent average ± SD of three independent experiments. (D–G) Increase in phosphorylation of ERK (D), JNK (E), p38 (F) and MSK-1 (G). After induction of C2C12 myotubes for 15 minutes with veh, iso and/or TNF, cells were lysed in SDS sample buffer and analysed via Western blotting. Bar charts represent average ± SD of densitometric analysis of three independent experiments. A representative blot is shown. Statistical significance was determined via ANOVA followed by Bonferroni's multiple comparison test (*) Significantly different from veh. (***) Significantly different from iso.

Because isoproterenol enhanced the expression of several typical NF-κB target genes, we explored what could be the molecular basis of these effects. Upon binding to its receptor(s), TNF-α triggers the canonical NF-κB cascade, initiated by IKKβ activation, IκBα degradation, phosphorylation of the NF-κB p65 subunit on serine 536 and its subsequent translocation from the cytoplasm to the nucleus [4]. As evaluated by Western blotting, we detected canonical NF-κB activation in C2C12 myotubes treated with TNF-α. We observed rapid phosphorylation of p65 on serine 536, degradation of IκBα (Figure 3 A), and nuclear translocation of p65 (Figure 3B). In line with this, TNF-α also induced an NF-κB reporter gene transiently transfected in C2C12 cells (Figure 3C). Isoproterenol treatment by itself had no effect on any of these aspects of canonical NF-κB activation and did not modulate the effects of TNF-α (Figure 3A-C).

In addition to the NF-κB pathway, TNF-α triggers activation of Mitogen Activated Protein Kinases (MAPKs) and Mitogen and Stress-activated Kinase 1 (MSK-1) which cooperate with NF-κB in driving inflammation [17], [25]. We found that in C2C12 myotubes, TNF-α activated JNK (Jun N-terminal kinase), ERK (Extracellular signal-regulated kinase) and p38 MAPKs and MSK-1 (Figure 3D-G). Isoproterenol by itself did not have significant effects on the phosphorylation of any of the MAPKs or MSK-1. Isoproterenol slightly inhibited TNF-α-induced phosphorylation of ERK, JNK and MSK-1, whereas it did not significantly affect TNF-α-induced p38 MAPK activation (Figure 3D–G). Collectively, this suggests that the potentiation of TNF-α-induced gene expression by isoproterenol is unlikely to be mediated by cytoplasmic modulation of NF-κB activity (Figure 2).

**Figure 4:**
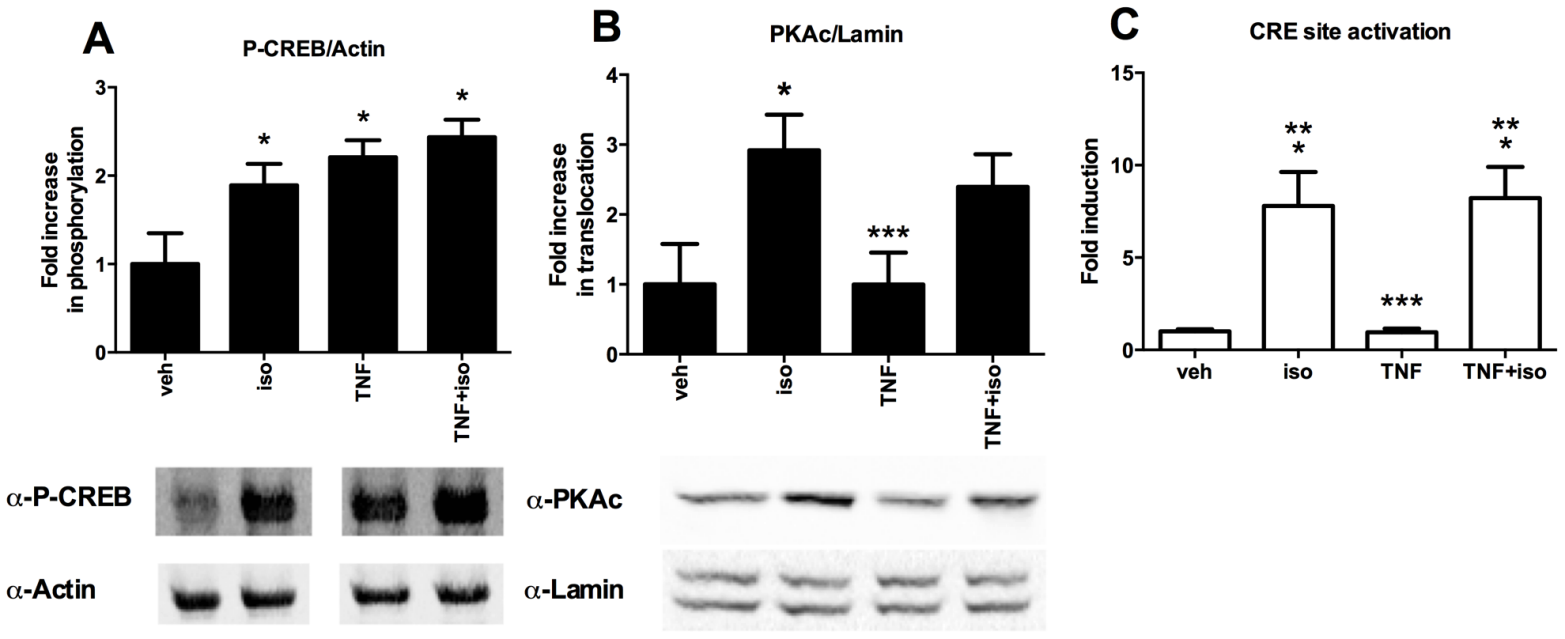
Effect of isoproterenol/TNF-α cotreatment on activation of the PKA/CREB cascade. (A) Increase in phosphorylation of CREB at serine 133. After induction of C2C12 myotubes for 15 minutes with veh, iso and/or TNF, cells were lysed in SDS sample buffer and analysed via Western blotting. Bar charts represent average ± SD of densitometric analysis of three independent experiments. A representative blot is shown. (B) Effect on nuclear translocation of PKAc. After veh, iso and/or TNF treatment for 15 min, nuclear extracts were prepared and analysed via Western blotting. Bar charts represent average ± SD of densitometric analysis of three independent experiments. A representative blot is shown. (C) Effect on the activation of CREB. C2C12 myotubes, transfected with the CRE-luciferase reporter plasmid, were treated with veh, TNF and/or iso for 6 hours, before analysis of luciferase production. Fold induction was calculated versus veh control. Results represent average ± SD of three independent experiments. Statistical significance was determined via ANOVA followed by Bonferroni's multiple comparison test (*) Significantly different from veh. (***) Significantly different from iso.

The canonical signalling pathway that is induced by β_2_-AR triggering is the protein kinase A (PKA)/ cAMP-response element-binding protein (CREB) cascade. We detected rapid phosphorylation of CREB on serine 133 in cells treated with isoproterenol as well as TNF-α. Combined treatment did not further potentiate CREB phosphorylation (Figure 4A). Nuclear translocation of the PKA catalytic subunit (PKAc) was only detected in cells treated with isoproterenol and not in TNF-α-treated cells (Figure 4B). Whereas both TNF-α and isoproterenol induced CREB phosphorylation, only isoproterenol-activated CREB was able to induce a CRE reporter gene in C2C12 cells (Figure 4C). TNF-α cotreatment did not modulate isoproterenol-induced CRE activity.

### β_2_-AR/TNF-R cotreatment induces chromatin modifications required for efficient recruitment of NF-κB

Because we did not detect any cytoplasmic β_2_-AR/TNF-Rs crosstalk events that could explain the observed synergy, we next explored nuclear crosstalk. We used the IL-6 gene, a prototypical NF-κB target gene, with known function in skeletal muscle, and the most potently affected gene in our RT-qPCR analysis, as a model system. The IL-6 gene has a complex promoter architecture and, among others, contains functional transcription factor binding sites for NF-κB and CREB [17], [26].

**Figure 5:**
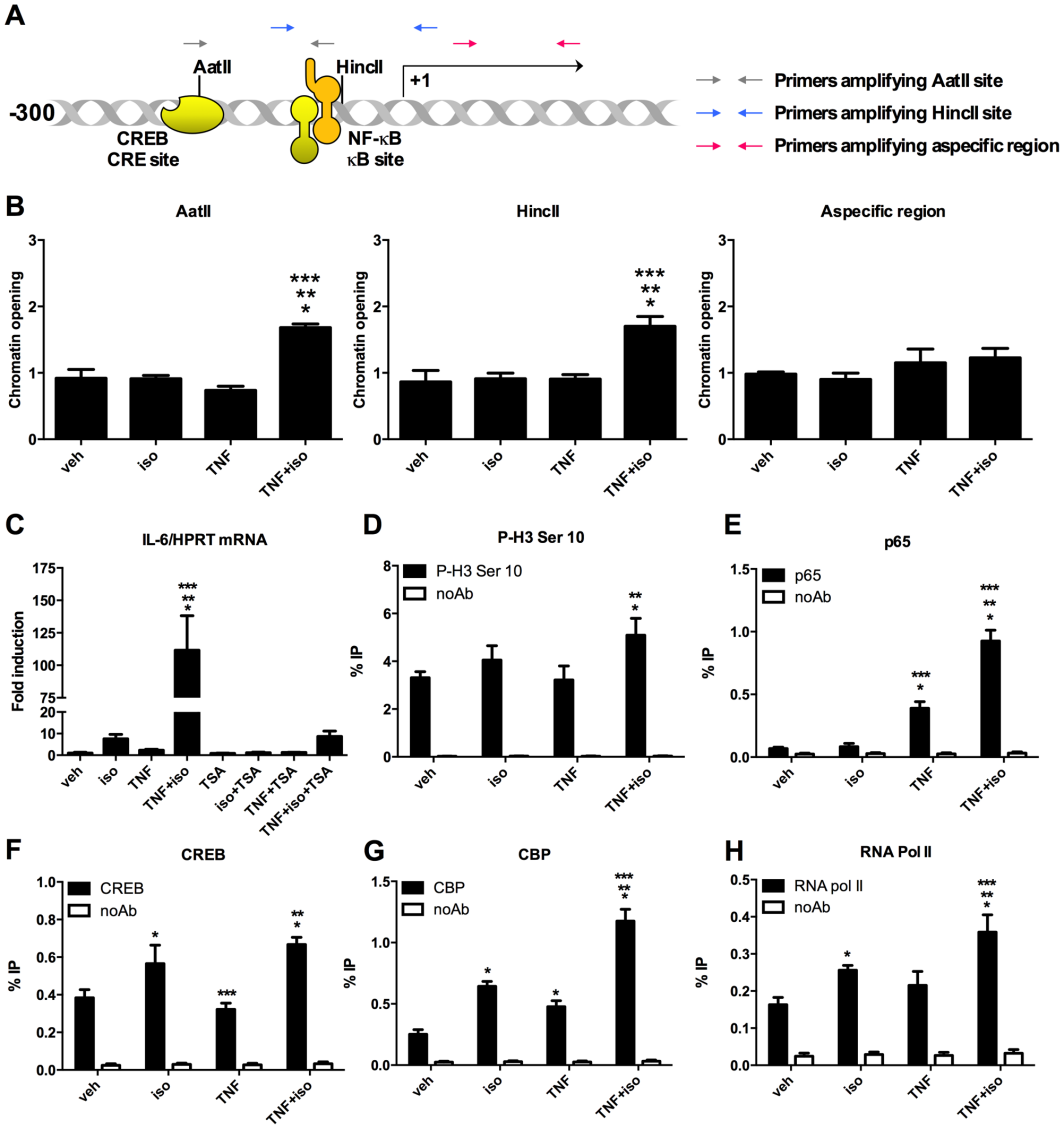
TNF-α/isoproterenol cotreatment induces chromatin remodelling and histone H3 modifications at the IL-6 promoter in C2C12 myotubes. (A) Schematic representation of the localisation of CREB- and NF-κB-responsive elements in the IL-6 promoter. Relative position of the recognition sites for AatII and HincII and primers used in the restriction enzyme accessibility assay via Real Time PCR (CHART-PCR) are indicated. (B) TNF/iso cotreatment promotes accessibility of the IL-6 promoter. C2C12 cells were treated for 2 hours with veh, iso and/or TNF. Chromatin opening of the promoter region was determined by CHART-PCR as detailed in Materials and Methods. Results represent average ± SD of three independent experiments. (C) Effect of TSA, a histone deacetylase inhibitor, on IL-6 transcription. C2C12 cells were treated for 2 hours with combinations of veh, TSA, iso and/or TNF. mRNA levels of IL-6 were determined via RT-qPCR. Results represent average ± SD of three independent experiments. (D) TNF/iso cotreatment enhances phosphorylation of histone H3 at the IL-6 promoter. C2C12 cells were treated with veh, iso and/or TNF for 2 hours. Phosphorylation of histone H3 at serine 10 was determined via ChIP. Results represent average ± SD of three independent experiments. (E-H) Effect of TNF and/or iso treatment on the recruitment of NF-κB, CREB, CBP and RNA polymerase II to the IL-6 promoter. C2C12 cells were treated with veh, iso and/or TNF for 2 hours. Recruitment of NF-κB p65, CREB, CBP and RNA polymerase II was measured via ChIP. Results represent average ± SD of three independent experiments. (*) Significantly different from veh. (**) Significantly different from TNF. (***) Significantly different from iso.

The accessibility of a gene's promoter to transcription factors is reflected in its susceptibility to nuclease digestion. In CHART-PCR, this feature is exploited and the accessibility of a selected DNA sequence is determined by digesting chromatin using nucleases and then quantifying the amount of remaining uncut gDNA in the digested chromatin sample via qPCR. Here, we digested the proximal IL-6 gene promoter using two restriction enzymes (AatII, HincII) that cut in the proximal IL-6 promoter, in the vicinity of CREB and NF-κB binding sites respectively. Then, we amplified the sequence of interest via qPCR, using primers that recognise the sequence flanking the restriction enzyme recognition sites (Figure 5A). Results were normalised as explained in the Materials and Methods section and expressed as chromatin opening. We found that the IL-6 promoter is susceptible to digestion with the AatII and HincII restriction enzymes, only upon cotreatment with TNF-α and isoproterenol (Figure 5B). As expected, no chromatin opening was apparent, at an irrelevant region (not containing restriction sites for AatII and HincII) of the IL-6 gene (Figure 5B).

Covalent modification of histone tails is a crucial event in the regulation of chromatin dynamics. In particular enhanced acetylation of histone H3 has been detected at transcriptionally active chromatin [27]. To investigate whether histone acetylation is implicated in IL-6 transcription induced by TNF-α/isoproterenol cotreatment, we first explored how the histone deacetylase (HDAC) inhibitor Trichostatin A (TSA) affects IL-6 transcription. Remarkably, we found that TSA, at subcytotoxic dose (100 nM), almost completely abrogated IL-6 transcription triggered by isoproterenol, TNF-α or the combination of both, indicating IL-6 transcription is inhibited, rather than enhanced by histone acetylation (Figure 5C). To assess histone H3 acetylation at the IL-6 promoter, we performed ChIP using an antibody recognising histone H3 acetylated at the Lys 27 residue, a modification for which the association with transcriptional activation is well established [28]-[30]. We found that, at the IL-6 promoter, histone H3 carried a constitutive acetylation mark at Lys 27, that was not significantly enhanced upon isoproterenol and/or TNF-α treatment (Figure S3A). We also failed to detect enhanced histone H3 acetylation using a pan acetyl-histone-H3 antibody (data not shown). The efficiency of the dose of TSA that we used to promote histone acetylation was confirmed by the detection of histone H3 Lys 27 acetylation in TSA-treated C2C12 cells by confocal microscopy (Figure S3B).

We found that isoproterenol stimulated PKA nuclear entry in C2C12 cells, while TNF-α promoted phosphorylation of MSK-1. Intriguingly, both PKA and MSK-1 have been shown to trigger phosphorylation of histone H3 on serine 10, a modification associated with chromatin relaxation and transcriptional activity [31], [32]. Via ChIP analysis, we detected very modest histone H3 phosphorylation at the IL-6 promoter upon isoproterenol stimulation that was significantly potentiated upon cotreatment of C2C12 cells with TNF-α (Figure 5D). Finally, we observed that isoproterenol promoted recruitment of CREB, CREB-binding protein (CBP) and RNA polymerase II to the IL-6 promoter, whereas TNF-α induced recruitment of NF-κB p65. Combined treatment with TNF-α and isoproterenol moreover potentiated the recruitment of p65, CBP and RNA polymerase II to the IL-6 promoter (Figure 5E-H). Isoproterenol-induced CREB recruitment was not modulated by TNF-α cotreatment. The specificity of the observed responses is indicated by the fact that we did not detect any of the observed responses at an irrelevant housekeeping gene (GAPDH) or using aspecific antibodies or beads only for the immunoprecipitation (Figure S3A, C-H).

These results show that only combined stimulation with TNF-α and isoproterenol leads to chromatin remodelling at the IL-6 promoter.

### Myotube-derived cytokines induce migration of myoblasts

**Figure 6:**
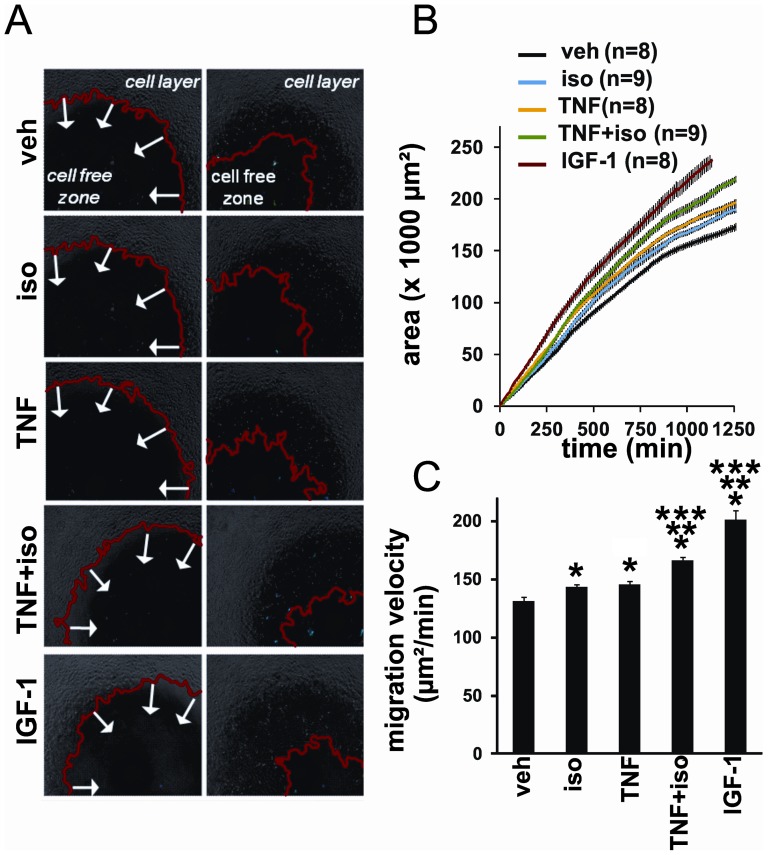
C2C12 myotubes secrete factors that promote the migration of C2C12 myoblasts. C2C12 myotubes were treated for 24/or TNF. Conditioned medium was prepared as described in Materials and Methods and applied to C2C12 myoblasts. Recombinant IGF-1 was used as a positive control. Cell migration was monitored for 24 hours. (A) Selected start and end phase contrast images for the different conditions from a representative experiment. The red line delineates the confluent cell layer that migrates inwards in time; arrows show migration direction into the cell-free zone. (B) Cell-covered area over time plot from a representative experiment. The lines represent the mean area for technical replicates (n indicated in the Figure); error bars are SEM. (C) Mean migration velocity (n replicates, see data in B) for the tested conditions in a representative experiment. (Relative migration efficiencies based on the cumulated data of three independent biological experiments are shown in Figure S4). Error bars are SEM. Statistical analysis was performed using Wilcoxon pairwise comparison with Bonferroni correction for multiple testing. (*) Significantly different from veh. (**) Significantly different from TNF. (***) Significantly different from iso.

Recent literature shows that, upon contraction or inflammation, skeletal muscle cells express several cytokines with chemotactic properties [24], [33]. Because we observed that cotreatment of C2C12 myotubes with isoproterenol enhanced transcription of several TNF-α-induced cyto-/chemokines that are known to modulate the migratory properties of cells, we investigated whether these factors induced migration of undifferentiated C2C12 myoblasts. It was reported that C2C12 myoblasts exhibit spontaneous mobility, which can be further stimulated by IGF-1 [33]. To analyse whether TNF-α/isoproterenol cotreatment induces the production of chemotactic factors by myotubes, we performed migration assays using myoblasts treated with myotube-conditioned medium. IGF-1 stimulation was used as a positive control. As shown in Figure 6 and Figure S4, myoblast cells treated with conditioned medium from myotubes stimulated with only TNF-α or isoproterenol displayed a modest, albeit significant increase in myoblast migration efficiency compared to the control condition. Importantly, myoblasts treated with conditioned medium derived from myotubes, which were stimulated with the combination of TNF-α and isoproterenol, migrated significantly faster than those treated with conditioned medium from myotubes treated with only TNF-α or isoproterenol (Figure 6C, Figure S4). This indicates that the conditioned medium of the cotreated myotubes has a stronger chemotactic potential on undifferentiated myoblasts.

## Discussion

The devastating role of TNF-α and NF-κB in skeletal muscle inflammation and atrophy is well documented [2], [5]. Here, we investigated whether and how β-agonists, that are known for their anabolic and anti-inflammatory properties, modulate the effects of TNF-α on the expression of inflammatory factors in skeletal muscle cells.

We used the murine C2C12 cell line as *in vitro* model system and found that the expression of β_2_-ARs was much more prominent, both at the mRNA and protein level, in differentiated myotubes as compared to undifferentiated myoblasts, indicating myotubes would be more susceptible to regulation by β_2_-AR-agonists. In agreement with this finding, elevated expression of the β_2_-AR was reported in regenerating myofibers after injury [7]. Whereas we also observed increased TNF-R1 expression at the mRNA level, this was not reflected at the protein level. Enhanced TNF-R1 mRNA levels were previously reported in regenerating myofibers, but the level of protein in the latter study was not investigated [34]. In another study decreased activation of NF-κB upon TNF-α treatment was observed upon myogenesis [35], but in this study TNF-R1 expression levels were not investigated and the diminished response could also be due to post-receptor regulatory mechanisms [36]. Our data suggest that in C2C12 cells TNF-R1 protein levels are not regulated upon myogenic differentiation, despite clear differences in mRNA levels. The reason for this discrepancy remains to be elucidated, but probably depends on post-transcriptional regulation of TNF-R1 expression.

Several proinflammatory mediators are upregulated in myotubes upon TNF-α challenge [15], [24] and in muscle wasting disorders [24], [37]. In line with this, we found a significant increase in the expression of selected NF-κB-dependent target genes in C2C12 cells treated with TNF-α. Interestingly, we observed that isoproterenol treatment by itself did not significantly affect the expression of these genes, but potently enhanced TNF-α-induced expression of a subset of inflammatory factors, such as IL-6 and CXCL5, and to a lesser extent that of CCL2 and ICAM-1, whereas that of others (CCL5 and IκBα) remained unaffected. The regulation of IL-6 expression was investigated previously in C2C12 cells stimulated with lipopolysaccharide (LPS) and epinephrine [13]. Similar to what we observe here using TNF-α as a proinflammatory stimulus, the authors reported synergistic IL-6 expression upon LPS/epinephrine cotreatment, indicating the synergy depends on a signalling protein that is common to TNF-R and Toll-like receptor (TLR) pathways. As opposed to previous reports of anti-inflammatory effects of β-agonists [10]–[12], we did not find any evidence for that in C2C12 cells. The anti-inflammatory effects of β-agonists have been mostly explained by β_2_-AR-mediated upregulation of IκBα levels, either via induction of its transcription, or by promoting its stability through interaction with the GPCR adaptor β-arrestin [10], [12], [38]. In C2C12 cells, we and others [13] did not detect any modulation of IκBα mRNA or protein levels, indicating β_2-_AR-dependent regulation of IκBα levels is a cell type-specific process. The lack of effects on IκBα expression was confirmed by our observation that isoproterenol cotreatment did not hamper nuclear translocation of NF-κB p65. In addition, we did not find potentiation of TNF-α-induced MAPKs or MSK-1 activation by isoproterenol, rather we found mild inhibition of MSK-1, ERK and JNK – indicating the effect of isoproterenol cannot be explained by cytoplasmic crosstalk with canonical TNF-α-induced signalling cascades. Whereas Frost *et al*. [13] suggested that epinephrine promotes activation of ERK, p38 and JNK MAPKs, we did not find any evidence for that using isoproterenol as a stimulus. Activation of these MAPKs by epinephrine in the publication by Frost *et al.* was however solely supported by effects of pharmacological inhibitors on IL-6 expression.

The most important novel finding in this paper is the observation that, in addition to IL-6, the expression of several chemokine genes is potentiated upon TNF-α/isoproterenol cotreatment of skeletal muscle cells. The genes we investigated in this study all contain NF-κB responsive elements in their proximal promoters. As is evident from Table S2, there is significant variation in the sequences of these elements. Although the investigated selection of genes is too small to make any reliable predictions, it is noteworthy that the NF-κB consensus sequences in the most responsive IL-6 (GGGATTTTCC) and CXCL5 (GGGAATTTCC) promoters bear the strongest similarity among the investigated genes. Recently, Siggers *et al.* [39] performed a comprehensive *in vitro* analysis of DNA binding by NF-κB dimers. Using Siggers' open source dataset, we evaluated whether the IL-6 and CXCL5 NF-κB sequences showed a particular preference for selected NF-κB dimers, as compared to genes that were not prone to TNF-α/isoproterenol coregulation, but could not find evidence for that. In fact, the binding preference of the NF-κB sequence in the IL-6 promoter resembled most that of the κB sequence in the IκBα promoter, which was not susceptible to synergistic regulation by TNF-α/isoproterenol. Evaluating whether the sequence of the NF-κB responsive element determining for the response to isoproterenol will however require more extensive, genome-wide, analysis. In addition to specifying NF-κB dimer binding, the sequence of the κB site also dictates which co-activators will bind to a selected promoter, and accumulating evidence suggests NF-κB dimers can adopt different conformations when bound to different DNA sequences [40]. In line with this, even a single nucleotide change within a κB sequence was shown to affect cofactor specificity of NF-κB dimers [41]. Cofactor specificity is also determined by post-translational modifications of NF-κB family members. Interestingly, both PKA and MSK-1 have been shown to phosphorylate p65 on its serine 276 residue, which promotes its association with the CBP coactivator and selective NF-κB dependent gene induction [42], [43]. Because of the lack of specific antibodies recognizing p65 phosphorylated at the serine 276 residue, we were unable to investigate whether this phosphorylation is triggered in C2C12 cells upon activation of PKA and/or MSK-1 [44]. We recently also reported synergistic IL-6 expression in human 1321N1 astrocytes cotreated with TNF-α/isoproterenol [26] indicating the TNF-R1/β_2_-AR synergy is not a phenomenon restricted to C2C12 cells. Nevertheless, in astrocytes we also detected inhibitory effects of isoproterenol (for instance at the ICAM-1 gene), whereas in the current study isoproterenol did not block the expression of any of the investigated genes (including ICAM-1). Our results indicate that the effects of isoproterenol are not only gene-selective, with only a selection of NF-κB dependent genes being susceptible to potentiation by isoproterenol, but that there is also an important cell type-specific component determining the outcome of TNF-R1/β_2_-AR co-activation. This cell-type specificity is supported by other reports describing different effects of β-agonists on the expression of selected inflammatory mediators, depending on the cell type investigated [11], [45], [46].

Expression of NF-κB target genes is regulated at multiple levels and selectivity is accomplished, among others, by co-operation of multiple transcription factors and cofactors in so-called enhanceosome structures as well as by epigenetic mechanisms [47]. We showed via chromatin accessibility assays that β_2_-AR/TNF-R co-activation induces chromatin remodelling at the IL-6 promoter. Both MSK-1 and PKA can directly phosphorylate histone H3 and recruit chromatin-remodelling complexes leading to increased promoter accessibility [31], [32], [48]. In agreement with this, we observed that in C2C12 cells concurrent activation of MSK-1 and PKA is associated with enhanced histone H3 phosphorylation and chromatin relaxation at the IL-6 promoter. Whereas we cannot exclude that there are also effects of the individual triggers (as is indicated by detectable recruitment of p65, CREB, CBP and RNA polymerase II also upon stimulation of cells with only isoproterenol or TNF-α) that are below the detection limit of our assays, it is clear that β_2_-AR/TNF-R co-activation promotes transcriptional synergy.

Intriguingly, it was reported that at the c-fos promoter (*in vitro*) CREB binding and phosphorylation are required to recruit MSK-1, which then phosphorylates histone H3 [49]. Here, we observed that, although both TNF-α and isoproterenol stimulate phosphorylation of CREB on serine 133, CREB activation is only promoted in the presence of isoproterenol. These results are in agreement with previous reports demonstrating that both MSK-1 and PKC phosphorylate CREB on serine 133 to a similar extent as PKA, but that only PKA-mediated phosphorylation resulted in CREB-dependent transcription [50], [51]. According to the generally accepted model for CREB-dependent transcriptional activation, CREB is constitutively bound to its target gene promoters and transcriptional activation requires phosphorylation of CREB. We have attempted to immunoprecipitate phosphorylated CREB from the IL-6 promoter using two antibodies that recognise CREB phosphorylated at serine 133, but failed to recover it (data not shown). Instead, we found that CREB was actively recruited to the IL-6 promoter upon isoproterenol, but not upon TNF-α treatment. We previously reported CREB recruitment at the IL-6 promoter upon TNF/isoproterenol cotreatment in human astrocytes [26]. These findings are moreover in line with other reports demonstrating active CREB recruitment at selected gene promoters. For instance, it has been shown that CREB recruitment occurs at glucagon-responsive gene promoters in hepatocytes treated with forskolin [52], at the c-fos promoter in neurons treated with BDNF [53] and at the ICER promoter in forskolin-treated PC12 cells [54].

We found that TNF-α induced CREB phosphorylation, but did not promote activation of a CREB-dependent reporter gene and did not induce CREB recruitment to the IL-6 promoter. It is, however, possible that TNF-α-induced CREB phosphorylation plays a role in the transcriptional activation of other genes, perhaps requiring different CREB co-activators. Evaluating the contribution of TNF-α induced MSK-1 versus isoproterenol-induced PKA in CREB-mediated gene transcription will, however, require further study.

In addition to phosphorylation, acetylation of histones plays a crucial role in gene expression [27], [28]. Moreover, combinations of multiple histone modifications can reinforce the robustness of the chromatin state and as a consequence modulate gene expression [55], [56]. We previously proposed that the selective synergy at the IL-6 promoter in astrocytes was the result of co-operative recruitment of the transcriptional coactivator CBP by CREB and NF-κB, that are positioned in close proximity at the IL-6 promoter [26]. Interestingly, also the CXCL5 promoter contains a CREB and NF-κB element in its proximal promoter, indicating this could be a signature of genes susceptible to TNF-R1/β_2_-AR co-activation. CBP acts as a transcriptional coactivator by either acetylating histone tails via its intrinsic acetyl-transferase activity or by recruiting additional histone acetyl-transferases (HATs), hence promoting chromatin accessibility [57], [58]. Surprisingly, whereas CBP is efficiently recruited to the IL-6 promoter upon TNF-α/isoproterenol cotreatment in the present study, we did not find evidence for a positive role of histone acetylation in promoting chromatin relaxation. This observation is in line with a previous report showing reduced IL-6 expression in C2C12 cells treated with TSA [13] and suggests the existence of cell-type specific epigenetic regulatory mechanisms acting at the IL-6 promoter. Importantly, whereas previous studies show coupling of histone H3 phosphorylation and acetylation as prerequisite for transcriptional activation [59], [60], we could not find evidence for that in our model system. It could be that, at the IL-6 promoter, CBP has a function independent of its HAT activity. It is indeed well known that CBP can acetylate non-histone proteins and in this way affect transcriptional activity. For instance, it has been shown that CBP acetylates CREB [61] and NF-κB p65 [62], [63], and that these modifications are instrumental for efficient transcriptional activation. Further experiments will however be required to investigate whether this indeed occurs at the IL-6 promoter.

In conclusion, the most striking observation of this study is that co-activation of the TNF-R1 and β_2_-AR potently enhances the expression of a subset of inflammatory mediators in C2C12 skeletal muscle cells. Recent reports demonstrated that IL-6, as well as C-C and C-X-C chemokines, regulate various stages of skeletal muscle regeneration [33], [64], [65]. For example, IL-6, CXCL5, CCL2 and CCL5 were shown to induce leukocyte influx to remove damaged myofibers [24], [65] and stimulate myoblast migration to fill in damaged areas [33], [64], [66]. Here, we show that cotreatment of myotubes with TNF-α and isoproterenol synergistically promotes the secretion of factors that induce the migration of undifferentiated myoblasts *in vitro*. In future studies, it would be interesting to investigate how this affects the recruitment of satellite cells and leukocytes *in vivo*, and in particular in models of TNF-α-dependent muscle disease.

**Figure 7:**
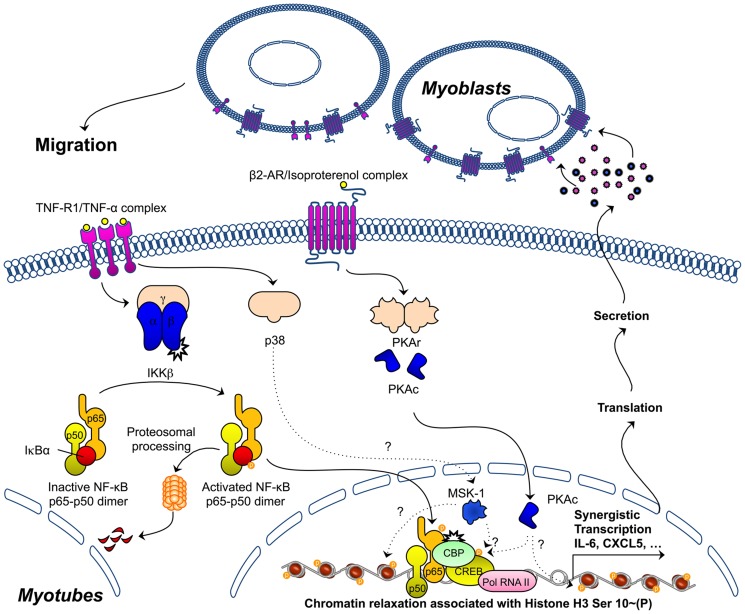
Model describing signalling events that are associated with β2-AR/TNFR coactivation in C2C12 cells, ultimately leading to transcriptional synergy at selected NF-κB-dependent promoters. For a detailed description we refer to the text in the discussion. Dotted lines indicate connections that are based on correlations and hence should be interpreted as assumptions rather than proven facts.

Based on our findings, we propose the following model (Figure 7): in C2C12 myotubes (I) TNF-R activation promotes activation of the canonical NF-κB signalling cascade and MAPKs, including the downstream p38 MAPK target MSK-1, which can phosphorylate CREB (II) β_2_-AR activation is associated with activation of PKA, which is the canonical CREB kinase (III) β_2_-AR activation does not interfere with early events in TNF-triggered pro-inflammatory NF-κB and MAPK activation (IV) β_2_-AR/TNF-R coactivation is associated with enhanced phosphorylation of histone H3 and chromatin relaxation at selected gene promoters (i.e. IL-6) and this is paralleled by recruitment of CREB, NF-κB p65 and CBP transcriptional coactivators as well as RNA polymerase II (V) TNF-R/β_2_-AR coactivation leads to synergistic gene expression of selected pro-inflammatory mediators and the secretion of factors that promote the migration of undifferentiated myoblasts.

Finally, as we did not detect anti-inflammatory effects of β-agonists in C2C12 skeletal muscle cells, but instead potentiation of TNF-α action, we suggest caution in the proposed use of β-agonists as therapeutic agents for inflammatory myopathies and urge for further study.

## Supporting Information

Figure S1 Myogenesis correlates with the expression of myogenin (Myog) in C2C12 skeletal muscle cells. The basal expression patterns of myogenin (Myog) mRNA (A) and protein (B) were compared in C2C12 myoblasts versus myotubes using RT-qPCR and Western blotting. For Western blotting, cells were lysed in RIPA buffer. A representative blot from two independent experiments is shown. (C) Confirmation of the β_2_-AR antibody specificity. Increasing amounts of an expression plasmid encoding a haemagglutinin-tagged β2-AR-HA (β2-AR-HA) was transiently transfected in HEK 293T cells. Cells were lysed in SDS sample buffer and analysed via Western blotting using α-β_2_-AR and/or α-HA. A representative blot from two independent experiments is shown.
(TIF)

Figure S2 Effect of β-agonist cotreatment on NF-κB-dependent gene expression in C2C12 myotubes. Expression of muscle-derived cytokines was measured by RT-qPCR after 2, 5 and 16-hours induction with veh, iso and/or TNF in C2C12 myotubes. Fold induction for each gene was calculated versus veh control at the corresponding time point. Results represent average ± SD of three independent experiments.
(TIF)

Figure S3 Nuclear events associated with iso/TNF cotreatment. (A) ChIP analysis of histone H3 acetylation. C2C12 cells were treated with vehicle or iso/TNF for up to 2 hours. Kinetics of histone H3 acetylation were determined via ChIP using an antibody recognizing histone H3 acetylated at Lys 27. Results represent average ± SD of three independent experiments. (B) TSA treatment promotes global histone H3 acetylation at Lys 27 in C2C12 cells. The efficiency of TSA in promoting histone acetylation was checked via confocal microscopy using anti-acetyl Lys 27 histone H3. The scale bar in the image equals 5 µM. The experiment was performed three times and a representative image is shown. (C–G) Control of ChIP assay gene specificity. Control ChIP experiments showing specificity of the observed responses for the IL-6 promoter. ChIP samples from the experiments shown in Figure 5 were re-analyzed using primers amplifying the GAPDH housekeeping gene promoter. Results represent average ± SD of three independent experiments. Statistical signifance was determined via ANOVA followed by Bonferroni's multiple comparison test. (*) Significantly different from veh. (**) Significantly different from TNF. (***) Significantly different from iso. (H) ChIP aspecific antibody background control. Control ChIP experiments comparing signals obtained using protein A beads only or aspecific control antibodies (normal rabbit IgG or normal goat IgG) versus signals obtained using selected specific antibodies (CREB and p65). Cells were treated for 2 hrs with iso+TNF, which is the optimal time point for detection of CREB and p65 recruitment. Results represent average ± SD of three independent experiments.
(TIF)

Figure S4 C2C12 myotubes secrete factors that promote migration of C2C12 myoblasts. C2C12 myotubes secrete factors that promote the migration of C2C12 myoblasts. Relative migration efficiency based on the cumulated data of three independent biological experiments (total number of technical replicates, n = 22,24,23,25,25 for veh, iso, TNF, TNF/iso and IGF-1 respectively). Migration efficiency is a measure of the ratio of the obtained velocity versus the one in ‘veh’ condition. Statistical analysis was performed using Wilcoxon pairwise comparison with Bonferroni correction for multiple testing. (*) Significantly different from veh. (**) Significantly different from TNF. (***) Significantly different from iso. The table shows p-values indicating the result of pairwise comparison of the indicated conditions. Only ‘TNF’ and ‘iso’ do not significantly differ in migration efficiency (p>0.05).
(TIF)

Table S1 Summary of primer sequences used in the present study.
(DOCX)

Table S2 Summary of the position and sequence of NF-κB binding sites in the proximal promoters of selected genes.
(DOCX)

## References

[1] Peterson JM, Guttridge DC (2008) Skeletal muscle diseases, inflammation, and NF-kappaB signaling: insights and opportunities for therapeutic intervention. Int Rev Immunol 27: 375–387. 18853344 10.1080/08830180802302389

[2] Li YP, Reid MB (2001) Effect of tumor necrosis factor-alpha on skeletal muscle metabolism. Curr Opin Rheumatol 13: 483–487. 11698724 10.1097/00002281-200111000-00005

[3] Dempsey PW, Doyle SE, He JQ, Cheng G (2003) The signaling adaptors and pathways activated by TNF superfamily. Cytokine Growth Factor Rev 14: 193–209. 12787559 10.1016/s1359-6101(03)00021-2

[4] Hayden MS, Ghosh S (2012) NF-kappaB, the first quarter-century: remarkable progress and outstanding questions. Genes Dev 26: 203–234. 22302935 10.1101/gad.183434.111 PMC3278889

[5] Mourkioti F, Rosenthal N (2008) NF-kappaB signaling in skeletal muscle: prospects for intervention in muscle diseases. J Mol Med (Berl) 86: 747–759. 18246321 10.1007/s00109-008-0308-4 PMC2480606

[6] Lynch GS, Ryall JG (2008) Role of beta-adrenoceptor signaling in skeletal muscle: implications for muscle wasting and disease. Physiol Rev 88: 729–767. 18391178 10.1152/physrev.00028.2007

[7] Beitzel F, Gregorevic P, Ryall JG, Plant DR, Sillence MN, et al (2004) Beta2-adrenoceptor agonist fenoterol enhances functional repair of regenerating rat skeletal muscle after injury. J Appl Physiol 96: 1385–1392. 14607853 10.1152/japplphysiol.01081.2003

[8] Ryall JG, Gregorevic P, Plant DR, Sillence MN, Lynch GS (2002) Beta 2-agonist fenoterol has greater effects on contractile function of rat skeletal muscles than clenbuterol. Am J Physiol Regul Integr Comp Physiol 283: R1386–1394. 12388476 10.1152/ajpregu.00324.2002

[9] Hinkle RT, Hodge KM, Cody DB, Sheldon RJ, Kobilka BK, et al (2002) Skeletal muscle hypertrophy and anti-atrophy effects of clenbuterol are mediated by the beta2-adrenergic receptor. Muscle Nerve 25: 729–734. 11994968 10.1002/mus.10092

[10] Farmer P, Pugin J (2000) beta-adrenergic agonists exert their “anti-inflammatory” effects in monocytic cells through the IkappaB/NF-kappaB pathway. Am J Physiol Lung Cell Mol Physiol 279: L675–682. 11000127 10.1152/ajplung.2000.279.4.L675

[11] Ye RD (2000) beta-Adrenergic agonists regulate NF-kappaB activation through multiple mechanisms. Am J Physiol Lung Cell Mol Physiol 279: L615–617. 11000119 10.1152/ajplung.2000.279.4.L615

[12] Gavrilyuk V, Dello Russo C, Heneka MT, Pelligrino D, Weinberg G, et al (2002) Norepinephrine increases I kappa B alpha expression in astrocytes. J Biol Chem 277: 29662–29668. 12050158 10.1074/jbc.M203256200

[13] Frost RA, Nystrom GJ, Lang CH (2004) Epinephrine stimulates IL-6 expression in skeletal muscle and C2C12 myoblasts: role of c-Jun NH2-terminal kinase and histone deacetylase activity. Am J Physiol Endocrinol Metab 286: E809–817. 14722032 10.1152/ajpendo.00560.2003

[14] Steensberg A, Toft AD, Schjerling P, Halkjaer-Kristensen J, Pedersen BK (2001) Plasma interleukin-6 during strenuous exercise: role of epinephrine. Am J Physiol Cell Physiol 281: C1001–1004. 11502577 10.1152/ajpcell.2001.281.3.C1001

[15] Bhatnagar S, Panguluri SK, Gupta SK, Dahiya S, Lundy RF, et al (2010) Tumor necrosis factor-alpha regulates distinct molecular pathways and gene networks in cultured skeletal muscle cells. PLoS One 5: e13262. 20967264 10.1371/journal.pone.0013262 PMC2953497

[16] Spurlock DM, McDaneld TG, McIntyre LM (2006) Changes in skeletal muscle gene expression following clenbuterol administration. BMC Genomics 7: 320. 17181869 10.1186/1471-2164-7-320 PMC1766935

[17] Vanden Berghe W, Plaisance S, Boone E, De Bosscher K, Schmitz ML, et al (1998) p38 and extracellular signal-regulated kinase mitogen-activated protein kinase pathways are required for nuclear factor-kappaB p65 transactivation mediated by tumor necrosis factor. J Biol Chem 273: 3285–3290. 9452444 10.1074/jbc.273.6.3285

[18] Yaffe D, Saxel O (1977) Serial passaging and differentiation of myogenic cells isolated from dystrophic mouse muscle. Nature 270: 725–727. 563524 10.1038/270725a0

[19] Blau HM, Chiu CP, Webster C (1983) Cytoplasmic activation of human nuclear genes in stable heterocaryons. Cell 32: 1171–1180. 6839359 10.1016/0092-8674(83)90300-8

[20] Terrillon S, Bouvier M (2004) Roles of G-protein-coupled receptor dimerization. EMBO Rep 5: 30–34. 14710183 10.1038/sj.embor.7400052 PMC1298963

[21] Bockaert J, Pin JP (1999) Molecular tinkering of G protein-coupled receptors: an evolutionary success. EMBO J 18: 1723–1729. 10202136 10.1093/emboj/18.7.1723 PMC1171258

[22] Yoon JH, Song P, Jang JH, Kim DK, Choi S, et al (2011) Proteomic analysis of tumor necrosis factor-alpha (TNF-alpha)-induced L6 myotube secretome reveals novel TNF-alpha-dependent myokines in diabetic skeletal muscle. J Proteome Res 10: 5315–5325. 22023146 10.1021/pr200573b

[23] Pedersen BK, Febbraio MA (2012) Muscles, exercise and obesity: skeletal muscle as a secretory organ. Nat Rev Endocrinol 8: 457–465. 22473333 10.1038/nrendo.2012.49

[24] Porter JD, Guo W, Merriam AP, Khanna S, Cheng G, et al (2003) Persistent over-expression of specific CC class chemokines correlates with macrophage and T-cell recruitment in mdx skeletal muscle. Neuromuscul Disord 13: 223–235. 12609504 10.1016/s0960-8966(02)00242-0

[25] Vermeulen L, De Wilde G, Van Damme P, Vanden Berghe W, Haegeman G (2003) Transcriptional activation of the NF-kappaB p65 subunit by mitogen- and stress-activated protein kinase-1 (MSK1). EMBO J 22: 1313–1324. 12628924 10.1093/emboj/cdg139 PMC151081

[26] Spooren A, Kooijman R, Lintermans B, Van Craenenbroeck K, Vermeulen L, et al (2010) Cooperation of NFkappaB and CREB to induce synergistic IL-6 expression in astrocytes. Cell Signal 22: 871–881. 20100571 10.1016/j.cellsig.2010.01.018

[27] Kouzarides T (2007) Chromatin modifications and their function. Cell 128: 693–705. 17320507 10.1016/j.cell.2007.02.005

[28] Creyghton MP, Cheng AW, Welstead GG, Kooistra T, Carey BW, et al (2010) Histone H3K27ac separates active from poised enhancers and predicts developmental state. Proc Natl Acad Sci U S A 107: 21931–21936. 21106759 10.1073/pnas.1016071107 PMC3003124

[29] Tie F, Banerjee R, Stratton CA, Prasad-Sinha J, Stepanik V, et al (2009) CBP-mediated acetylation of histone H3 lysine 27 antagonizes Drosophila Polycomb silencing. Development 136: 3131–3141. 19700617 10.1242/dev.037127 PMC2730368

[30] Wang Z, Zang C, Rosenfeld JA, Schones DE, Barski A, et al (2008) Combinatorial patterns of histone acetylations and methylations in the human genome. Nat Genet 40: 897–903. 18552846 10.1038/ng.154 PMC2769248

[31] Salvador LM, Park Y, Cottom J, Maizels ET, Jones JC, et al (2001) Follicle-stimulating hormone stimulates protein kinase A-mediated histone H3 phosphorylation and acetylation leading to select gene activation in ovarian granulosa cells. J Biol Chem 276: 40146–40155. 11498542 10.1074/jbc.M106710200

[32] Drobic B, Perez-Cadahia B, Yu J, Kung SK, Davie JR (2010) Promoter chromatin remodeling of immediate-early genes is mediated through H3 phosphorylation at either serine 28 or 10 by the MSK1 multi-protein complex. Nucleic Acids Res 38: 3196–3208. 20129940 10.1093/nar/gkq030 PMC2879512

[33] Nedachi T, Hatakeyama H, Kono T, Sato M, Kanzaki M (2009) Characterization of contraction-inducible CXC chemokines and their roles in C2C12 myocytes. Am J Physiol Endocrinol Metab 297: E866–878. 19622786 10.1152/ajpendo.00104.2009

[34] Zador E, Mendler L, Takacs V, de Bleecker J, Wuytack F (2001) Regenerating soleus and extensor digitorum longus muscles of the rat show elevated levels of TNF-alpha and its receptors, TNFR-60 and TNFR-80. Muscle Nerve 24: 1058–1067. 11439381 10.1002/mus.1110

[35] Guttridge DC, Albanese C, Reuther JY, Pestell RG, Baldwin AS Jr (1999) NF-kappaB controls cell growth and differentiation through transcriptional regulation of cyclin D1. Mol Cell Biol 19: 5785–5799. 10409765 10.1128/mcb.19.8.5785 PMC84428

[36] Wertz IE, Dixit VM (2008) Ubiquitin-mediated regulation of TNFR1 signaling. Cytokine Growth Factor Rev 19: 313–324. 18515172 10.1016/j.cytogfr.2008.04.014

[37] De Paepe B, Creus KK, De Bleecker JL (2007) Chemokine profile of different inflammatory myopathies reflects humoral versus cytotoxic immune responses. Ann N Y Acad Sci 1109: 441–453. 17785333 10.1196/annals.1398.050

[38] Witherow DS, Garrison TR, Miller WE, Lefkowitz RJ (2004) beta-Arrestin inhibits NF-kappaB activity by means of its interaction with the NF-kappaB inhibitor IkappaBalpha. Proc Natl Acad Sci U S A 101: 8603–8607. 15173580 10.1073/pnas.0402851101 PMC423241

[39] Siggers T, Chang AB, Teixeira A, Wong D, Williams KJ, et al (2012) Principles of dimer-specific gene regulation revealed by a comprehensive characterization of NF-kappaB family DNA binding. Nat Immunol 13: 95–102. 10.1038/ni.2151 PMC3242931 22101729

[40] Smale ST (2012) Dimer-specific regulatory mechanisms within the NF-kappaB family of transcription factors. Immunol Rev 246: 193–204. 22435556 10.1111/j.1600-065X.2011.01091.x

[41] Leung TH, Hoffmann A, Baltimore D (2004) One nucleotide in a kappaB site can determine cofactor specificity for NF-kappaB dimers. Cell 118: 453–464. 15315758 10.1016/j.cell.2004.08.007

[42] Zhong H, Voll RE, Ghosh S (1998) Phosphorylation of NF-kappa B p65 by PKA stimulates transcriptional activity by promoting a novel bivalent interaction with the coactivator CBP/p300. Mol Cell 1: 661–671. 9660950 10.1016/s1097-2765(00)80066-0

[43] Vermeulen L, De Wilde G, Notebaert S, Vanden Berghe W, Haegeman G (2002) Regulation of the transcriptional activity of the nuclear factor-kappaB p65 subunit. Biochem Pharmacol 64: 963–970. 12213593 10.1016/s0006-2952(02)01161-9

[44] Spooren A, Kolmus K, Vermeulen L, Van Wesemael K, Haegeman G, et al (2010) Hunting for serine 276-phosphorylated p65. J Biomed Biotechnol 2010: 275892. 20204068 10.1155/2010/275892 PMC2829628

[45] Kaur M, Holden NS, Wilson SM, Sukkar MB, Chung KF, et al (2008) Effect of beta2-adrenoceptor agonists and other cAMP-elevating agents on inflammatory gene expression in human ASM cells: a role for protein kinase A. Am J Physiol Lung Cell Mol Physiol. 295: L505–514. 10.1152/ajplung.00046.2008 18586957

[46] Baouz S, Giron-Michel J, Azzarone B, Giuliani M, Cagnoni F, et al (2005) Lung myofibroblasts as targets of salmeterol and fluticasone propionate: inhibition of alpha-SMA and NF-kappaB. Int Immunol 17: 1473–1481. 16210331 10.1093/intimm/dxh325

[47] Smale ST (2011) Hierarchies of NF-kappaB target-gene regulation. Nat Immunol 12: 689–694. 21772277 10.1038/ni.2070 PMC3169328

[48] Monroy MA, Ruhl DD, Xu X, Granner DK, Yaciuk P, et al (2001) Regulation of cAMP-responsive element-binding protein-mediated transcription by the SNF2/SWI-related protein, SRCAP. J Biol Chem 276: 40721–40726. 11522779 10.1074/jbc.M103615200

[49] Shimada M, Nakadai T, Fukuda A, Hisatake K (2010) cAMP-response element-binding protein (CREB) controls MSK1-mediated phosphorylation of histone H3 at the c-fos promoter in vitro. J Biol Chem 285: 9390–9401. 20089855 10.1074/jbc.M109.057745 PMC2843188

[50] Mayr BM, Canettieri G, Montminy MR (2001) Distinct effects of cAMP and mitogenic signals on CREB-binding protein recruitment impart specificity to target gene activation via CREB. Proc Natl Acad Sci U S A 98: 10936–10941. 11535812 10.1073/pnas.191152098 PMC58577

[51] Avni D, Ernst O, Philosoph A, Zor T (2010) Role of CREB in modulation of TNFalpha and IL-10 expression in LPS-stimulated RAW264.7 macrophages. Mol Immunol 47: 1396–1403. 20303596 10.1016/j.molimm.2010.02.015

[52] Wang Y, Inoue H, Ravnskjaer K, Viste K, Miller N, et al (2010) Targeted disruption of the CREB coactivator Crtc2 increases insulin sensitivity. Proc Natl Acad Sci U S A 107: 3087–3092. 20133702 10.1073/pnas.0914897107 PMC2840317

[53] Riccio A, Alvania RS, Lonze BE, Ramanan N, Kim T, et al (2006) A nitric oxide signaling pathway controls CREB-mediated gene expression in neurons. Mol Cell 21: 283–294. 16427017 10.1016/j.molcel.2005.12.006

[54] Cha-Molstad H, Keller DM, Yochum GS, Impey S, Goodman RH (2004) Cell-type-specific binding of the transcription factor CREB to the cAMP-response element. Proc Natl Acad Sci U S A 101: 13572–13577. 15342915 10.1073/pnas.0405587101 PMC518796

[55] Schreiber SL, Bernstein BE (2002) Signaling network model of chromatin. Cell 111: 771–778. 12526804 10.1016/s0092-8674(02)01196-0

[56] Fischle W, Wang Y, Allis CD (2003) Histone and chromatin cross-talk. Curr Opin Cell Biol 15: 172–183. 12648673 10.1016/s0955-0674(03)00013-9

[57] McManus KJ, Hendzel MJ (2001) CBP, a transcriptional coactivator and acetyltransferase. Biochem Cell Biol 79: 253–266. 11467739

[58] Holmqvist PH, Mannervik M (2013) Genomic occupancy of the transcriptional co-activators p300 and CBP. Transcription 4: 18–23. 23131664 10.4161/trns.22601 PMC3644037

[59] Cheung P, Tanner KG, Cheung WL, Sassone-Corsi P, Denu JM, et al (2000) Synergistic coupling of histone H3 phosphorylation and acetylation in response to epidermal growth factor stimulation. Mol Cell 5: 905–915. 10911985 10.1016/s1097-2765(00)80256-7

[60] Clayton AL, Mahadevan LC (2003) MAP kinase-mediated phosphoacetylation of histone H3 and inducible gene regulation. FEBS Lett 546: 51–58. 12829236 10.1016/s0014-5793(03)00451-4

[61] Lu Q, Hutchins AE, Doyle CM, Lundblad JR, Kwok RP (2003) Acetylation of cAMP-responsive element-binding protein (CREB) by CREB-binding protein enhances CREB-dependent transcription. J Biol Chem 278: 15727–15734. 12595525 10.1074/jbc.M300546200

[62] Sundar IK, Chung S, Hwang JW, Lapek JD Jr, Bulger M, et al (2012) Mitogen- and stress-activated kinase 1 (MSK1) regulates cigarette smoke-induced histone modifications on NF-kappaB-dependent genes. PLoS One 7: e31378. 22312446 10.1371/journal.pone.0031378 PMC3270039

[63] Chen LF, Mu Y, Greene WC (2002) Acetylation of RelA at discrete sites regulates distinct nuclear functions of NF-kappaB. EMBO J 21: 6539–6548. 12456660 10.1093/emboj/cdf660 PMC136963

[64] Serrano AL, Baeza-Raja B, Perdiguero E, Jardi M, Munoz-Canoves P (2008) Interleukin-6 is an essential regulator of satellite cell-mediated skeletal muscle hypertrophy. Cell Metab 7: 33–44. 18177723 10.1016/j.cmet.2007.11.011

[65] Warren GL, O'Farrell L, Summan M, Hulderman T, Mishra D, et al (2004) Role of CC chemokines in skeletal muscle functional restoration after injury. Am J Physiol Cell Physiol 286: C1031–1036. 15075201 10.1152/ajpcell.00467.2003

[66] Corti S, Salani S, Del Bo R, Sironi M, Strazzer S, et al (2001) Chemotactic factors enhance myogenic cell migration across an endothelial monolayer. Exp Cell Res 268: 36–44. 11461116 10.1006/excr.2001.5267

