# Correction: β-Agonists Selectively Modulate Proinflammatory Gene Expression in Skeletal Muscle Cells via Non-Canonical Nuclear Crosstalk Mechanisms

**DOI:** 10.1371/journal.pone.0287938

**Published:** 2023-06-27

**Authors:** Krzysztof Kolmus, Marleen Van Troys, Karlien Van Wesemael, Christophe Ampe, Guy Haegeman, Jan Tavernier, Sarah Gerlo

The start phase iso panel in Fig 6A is incorrect as it is an inadvertent duplication of the start phase veh panel. The updated Fig 6 is provided here in which the start phase iso panel is corrected.

**Fig 6:**
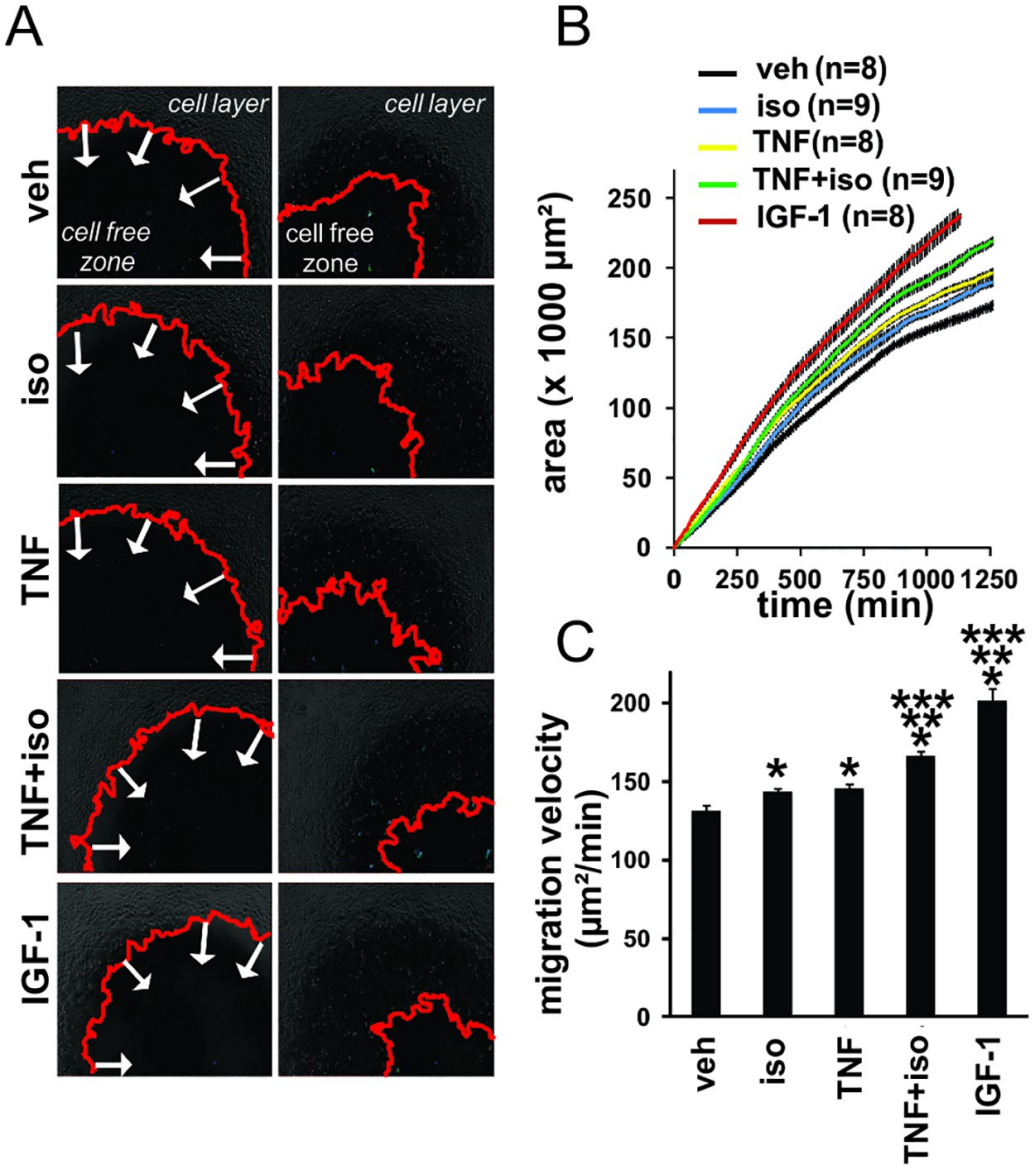
C2C12 myotubes secrete factors that promote the migration of C2C12 myoblasts. C2C12 myotubes were treated for 24 hrs with veh, iso, TNF and iso+TNF. Conditioned medium was prepared as described in Materials and Methods and applied to C2C12 myoblasts. Recombinant IGF-1 was used as a positive control. Cell migration was monitored for 24 hours. (A) Selected start and end phase contrast images for the different conditions from a representative experiment. The red line delineates the confluent cell layer that migrates inwards in time; arrows show migration direction into the cell-free zone. (B) Cell-covered area over time plot from a representative experiment. The lines represent the mean area for technical replicates (n indicated in the Figure); error bars are SEM. (C) Mean migration velocity (n replicates, see data in B) for the tested conditions in a representative experiment. (Relative migration efficiencies based on the cumulated data of three independent biological experiments are shown in Figure S4). Error bars are SEM. Statistical analysis was performed using Wilcoxon pairwise comparison with Bonferroni correction for multiple testing. (*) Significantly different from veh. (**) Significantly different from TNF. (***) Significantly different from iso. The experiment presented in this figure was performed independently three times, the images in Fig 6A are taken from one independent experiment and the results presented in Fig 6B and 6C are from a different independent experiment.

The figure legend has been updated to indicate that the experiment presented in Fig 6 was performed independently three times, the images in Fig 6A are taken from one independent experiment, and the results presented in Fig 6B and 6C are from a different independent experiment. The corresponding author confirms that the experimental conditions were identical for the images and data presented in Fig 6A–6C.

The raw data underlying the results in Fig 6B and 6C are provided here as S1 File. Representative videos of each condition in Fig 6 are provided in S2 File.

Additionally, the corresponding author has shared that, due to an irreparable hard disk failure the raw data underlying all figures except those for Fig 6 and Figure S4 are unavailable.

The corresponding author apologizes for the error in the published article.

## Supporting information

S1 File Raw data underlying the results in Fig 6B and 6C. (XLSX)

S2 File Representative videos of each condition in Fig 6. Representative videos for (A) veh, (B) iso, (C) TNF, (D) TNF + iso, and (E) IGF.
(ZIP)
