# Supplementary figures and images for: β-Agonists Selectively Modulate Proinflammatory Gene Expression in Skeletal Muscle Cells via Non-Canonical Nuclear Crosstalk Mechanisms
Source: PLoS One. 2014 Mar 6;9(3):e90649. doi: 10.1371/journal.pone.0090649 (PMC3946252; doi:10.1371/journal.pone.0090649)

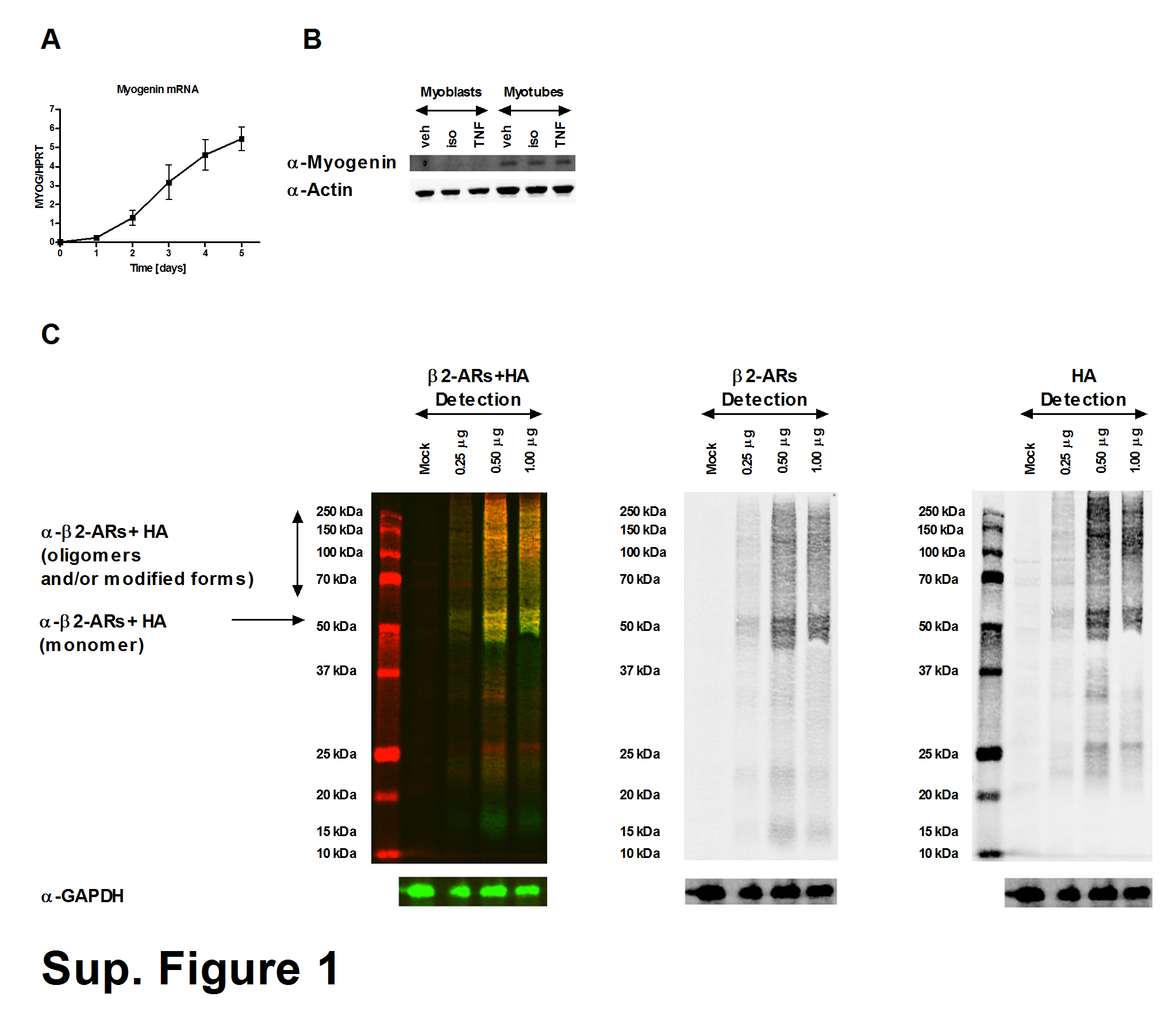

Supplement: Figure S1 — Myogenesis correlates with the expression of myogenin (Myog) in C2C12 skeletal muscle cells. The basal expression patterns of myogenin (Myog) mRNA (A) and protein (B) were compared in C2C12 myoblasts versus myotubes using RT-qPCR and Western blotting. For Western blotting, cells were lysed in RIPA buffer. A representative blot from two independent experiments is shown. (C) Confirmation of the β2-AR antibody specificity. Increasing amounts of an expression plasmid encoding a haemagglutinin-tagged β2-AR-HA (β2-AR-HA) was transiently transfected in HEK 293T cells. Cells were lysed in SDS sample buffer and analysed via Western blotting using α-β2-AR and/or α-HA. A representative blot from two independent experiments is shown. (TIF) [file pone.0090649.s001.tif]

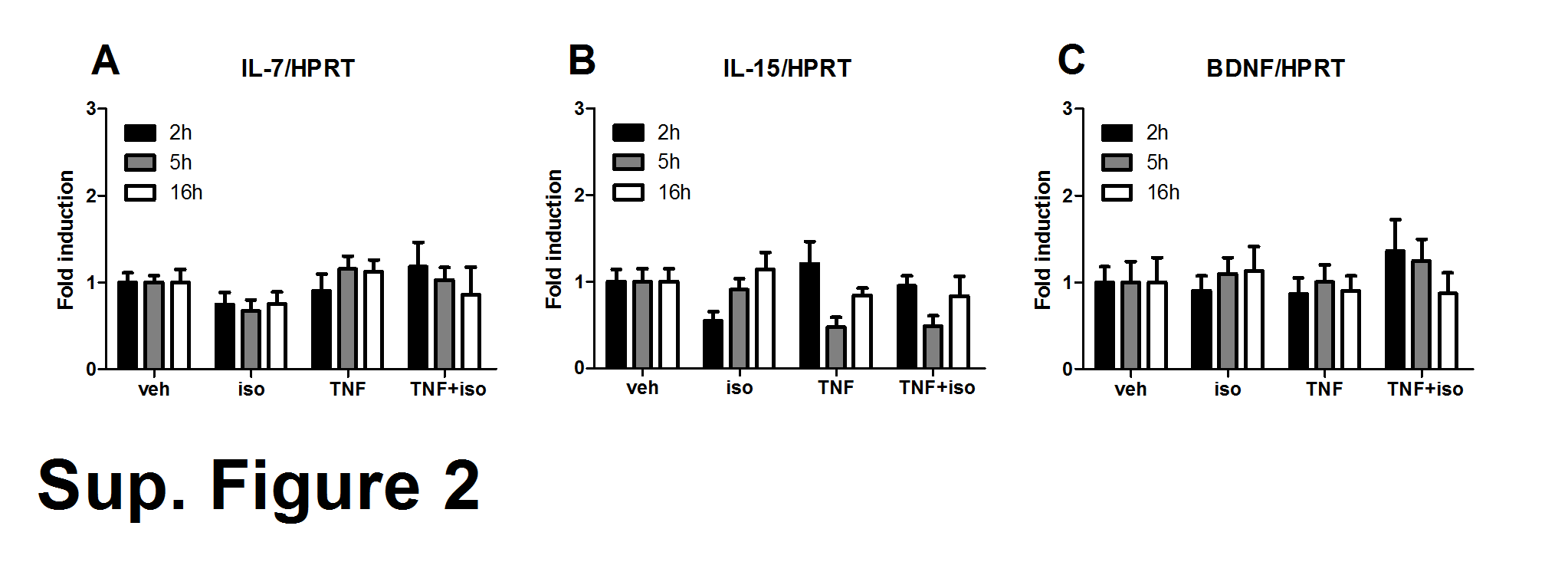

Supplement: Figure S2 — Effect of β-agonist cotreatment on NF-κB-dependent gene expression in C2C12 myotubes. Expression of muscle-derived cytokines was measured by RT-qPCR after 2, 5 and 16-hours induction with veh, iso and/or TNF in C2C12 myotubes. Fold induction for each gene was calculated versus veh control at the corresponding time point. Results represent average ± SD of three independent experiments. (TIF) [file pone.0090649.s002.tif]

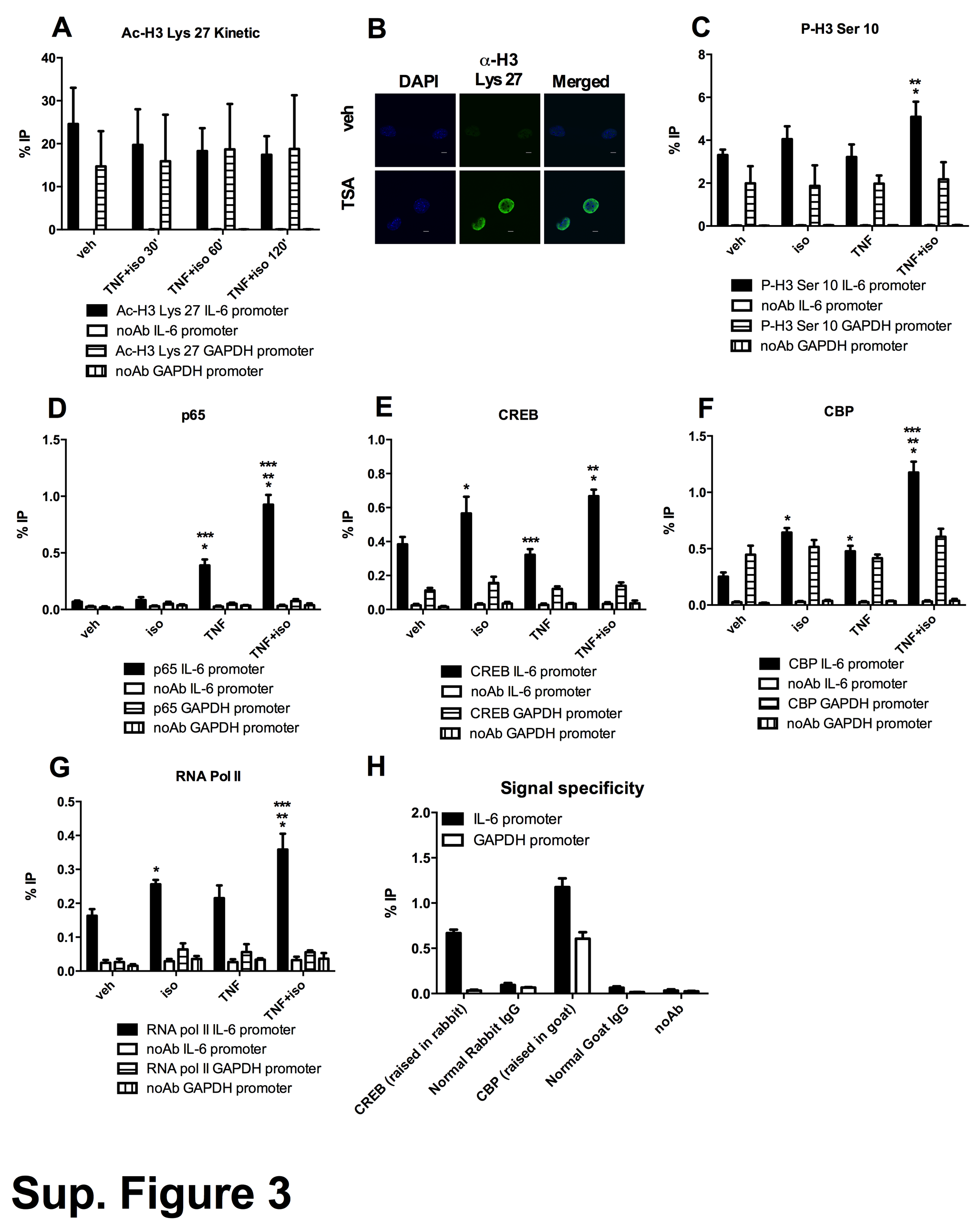

Supplement: Figure S3 — Nuclear events associated with iso/TNF cotreatment. (A) ChIP analysis of histone H3 acetylation. C2C12 cells were treated with vehicle or iso/TNF for up to 2 hours. Kinetics of histone H3 acetylation were determined via ChIP using an antibody recognizing histone H3 acetylated at Lys 27. Results represent average ± SD of three independent experiments. (B) TSA treatment promotes global histone H3 acetylation at Lys 27 in C2C12 cells. The efficiency of TSA in promoting histone acetylation was checked via confocal microscopy using anti-acetyl Lys 27 histone H3. The scale bar in the image equals 5 µM. The experiment was performed three times and a representative image is shown. (C–G) Control of ChIP assay gene specificity. Control ChIP experiments showing specificity of the observed responses for the IL-6 promoter. ChIP samples from the experiments shown in Figure 5 were re-analyzed using primers amplifying the GAPDH housekeeping gene promoter. Results represent average ± SD of three independent experiments. Statistical signifance was determined via ANOVA followed by Bonferroni's multiple comparison test. (*) Significantly different from veh. (**) Significantly different from TNF. (***) Significantly different from iso. (H) ChIP aspecific antibody background control. Control ChIP experiments comparing signals obtained using protein A beads only or aspecific control antibodies (normal rabbit IgG or normal goat IgG) versus signals obtained using selected specific antibodies (CREB and p65). Cells were treated for 2 hrs with iso+TNF, which is the optimal time point for detection of CREB and p65 recruitment. Results represent average ± SD of three independent experiments. (TIF) [file pone.0090649.s003.tif]

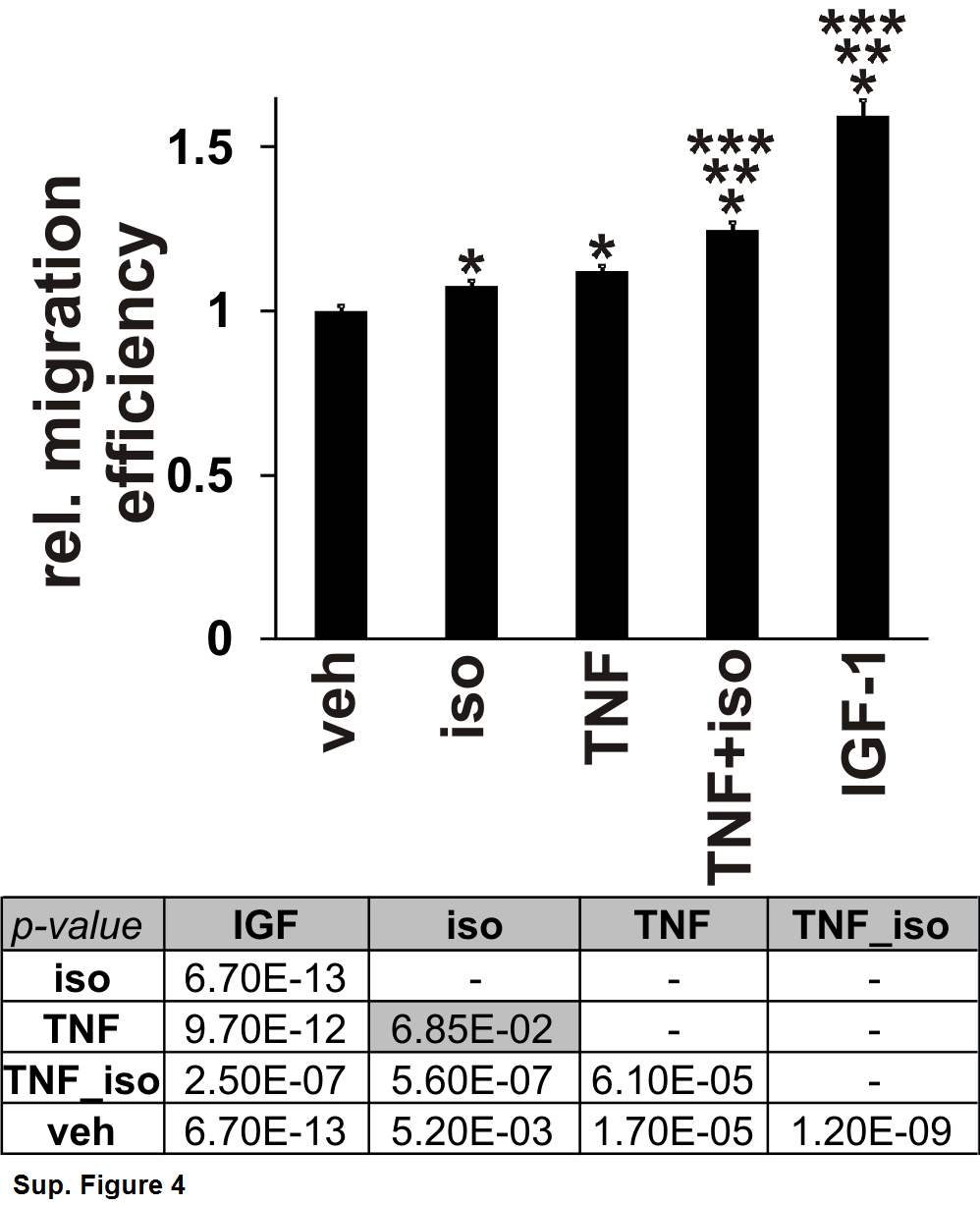

Supplement: Figure S4 — C2C12 myotubes secrete factors that promote migration of C2C12 myoblasts. C2C12 myotubes secrete factors that promote the migration of C2C12 myoblasts. Relative migration efficiency based on the cumulated data of three independent biological experiments (total number of technical replicates, n = 22,24,23,25,25 for veh, iso, TNF, TNF/iso and IGF-1 respectively). Migration efficiency is a measure of the ratio of the obtained velocity versus the one in ‘veh’ condition. Statistical analysis was performed using Wilcoxon pairwise comparison with Bonferroni correction for multiple testing. (*) Significantly different from veh. (**) Significantly different from TNF. (***) Significantly different from iso. The table shows p-values indicating the result of pairwise comparison of the indicated conditions. Only ‘TNF’ and ‘iso’ do not significantly differ in migration efficiency (p>0.05). (TIF) [file pone.0090649.s004.tif]
