# Supplementary material for: β-Agonists Selectively Modulate Proinflammatory Gene Expression in Skeletal Muscle Cells via Non-Canonical Nuclear Crosstalk Mechanisms
Source: PLoS One. 2014 Mar 6;9(3):e90649. doi: 10.1371/journal.pone.0090649 (PMC3946252; doi:10.1371/journal.pone.0090649)
Supplement: Table S1 — Summary of primer sequences used in the present study. (DOCX) [file pone.0090649.s005.docx]

**Supporting information tables**

**Table S1. Summary of primer sequences used in the present study.**

| **Name** |  |  | **Sequence** |  |
| --- | --- | --- | --- | --- |
| Hypoxanthine guanine phosphoribosyl transferase (HPRT) | fw | 5' | CCTAAGATGAGCGCAAGTTGAA | 3' |
|  | rv | 5' | CCACAGGACTAGAACACCTGCTAA | 3' |
| Interleukin-6 (IL-6) | fw | 5' | AGTCCTTCCTACCCCAATTTCC | 3' |
|  | rv | 5' | TTGGTCCTTAGCCACTCCTTC | 3' |
| Interleukin-7 (IL-7) | fw | 5' | GTGCTGCTCGCAAGTTGAAG | 3' |
|  | rv | 5' | AGTTCACCAGTGTTTGTGTGC | 3' |
| Interleukin-15 (IL-15) | fw | 5' | CATCCATCTCGTGCTACTTGTG | 3' |
|  | rv | 5' | GCCTCTGTTTTAGGGAGACCT | 3' |
| Brain-derived neurotrophic factor (BDNF) | fw | 5' | TCATACTTCGGTTGCATGAAGG | 3' |
|  | rv | 5' | AGACCTCTCGAACCTGCCC | 3' |
| Chemokine (C-C motif) ligand 2 (CCL2) | fw | 5' | TTAAAAACCTGGATCGGAACCAA | 3' |
|  | rv | 5' | GCATTAGCTTCAGATTTACGGGT | 3' |
| Chemokine (C-C motif) ligand 5 (CCL5) | fw | 5' | TTTGCCTACCTCTCCCTCG | 3' |
|  | rv | 5' | CGACTGCAAGATTGGAGCACT | 3' |
| Chemokine (C-X-C motif) ligand 5 (CXCL5) | fw | 5' | GTGTTTGCTTAACCGTAACTCCA | 3' |
|  | rv | 5' | CTTCCACCGTAGGGCACTG | 3' |
| Intercellular adhesion molecule1 (ICAM-1) | fw | 5' | CCGCAGGTCCAATTCACACT | 3' |
|  | rv | 5' | TCCAGCCGAGGACCATACAG | 3' |
| Nuclear factor of kappa B inhibitor α (IκBα) | fw | 5' | TGAAGGACGAGGAGTACGAGC | 3' |
|  | rv | 5' | TTCGTGGATGATTGCCAAGTG | 3' |
| Myogenin (MYOG) | fw | 5' | GGGCAATGCACTGGAGTTCG | 3' |
|  | rv | 5' | CAGATTGTGGGCGTCTGTAG | 3' |
| IL-6 NheI, CHART-PCR | fw | 5' | CGTGCATGACTTCAGCTTTAC | 3' |
|  | rv | 5' | TGCAGCTTAGGTCGTCATTG | 3' |
| IL-6 AatII, CHART-PCR, ChIP | fw | 5' | GCCTCAAGGATGACTTAAGC | 3' |
|  | rv | 5' | TGTGACGTCGTTTAGCATCG | 3' |
| IL-6 aspecific region, CHART-PCR | fw | 5' | ACCGCTATGAAGTTCCTCTC | 3' |
|  | rv | 5' | AACCCACAATGCTGGCTCTC | 3' |
| Glyceraldehyde 3-phosphate dehydrogenase (GAPDH) ChIP | fw | 5' | GATGCAGGGATGATGTTC | 3' |
|  | rv | 5' | TGCACCACCAACTGCTTAG | 3' |
