# Supplementary material for: β-Agonists Selectively Modulate Proinflammatory Gene Expression in Skeletal Muscle Cells via Non-Canonical Nuclear Crosstalk Mechanisms
Source: PLoS One. 2014 Mar 6;9(3):e90649. doi: 10.1371/journal.pone.0090649 (PMC3946252; doi:10.1371/journal.pone.0090649)
Supplement: Table S2 — Summary of the position and sequence of NF-κB binding sites in the proximal promoters of selected genes. (DOCX) [file pone.0090649.s006.docx]

**Table S2. Summary of the position and sequence of NF-κB binding sites in the proximal promoters of selected genes.**

| **Name** | **Position** |  | **Sequence** |  |
| --- | --- | --- | --- | --- |
| Interleukin-6 (IL-6) | -91 | 5' | GGGATTTTCC | 3' |
| Interleukin-7 (IL-7) | -24 | 5' | TCAGATCCCC | 3' |
| Interleukin-15 (IL-15) | -243 | 5' | CGCCTTGTTT | 3' |
| Brain-derived neurotrophic factor (BDNF) | -117 | 5' | AGAAGTTTCC | 3' |
| Chemokine (C-C motif) ligand 2 (CCL2) | -152 | 5' | GGAAACACCCG | 3' |
| Chemokine (C-C motif) ligand 5 (CCL5) | -90 | 5' | GGAAACTCCC | 3' |
| Chemokine (C-X-C motif) ligand 5 (CXCL5 ) | -29 | 5' | GGGAATTTCC | 3' |
| Intercellular adhesion molecule1 (ICAM-1) | -174 | 5' | TGGAAATTCC | 3' |
| Nuclear factor of kappa B inhibitor α (IκBα) | -29 | 5' | GGAAATTCCC | 3' |

Bioinformatics analysis of promoters was performed using P-Scan. A region of 500 bp upstream to the transcription starting site of the selected promoters was investigated with the Transcription Factor Binding Sites matrices from TRANSFAC databases.
